# Synthesis, characterization, antimicrobial, antioxidant, and antiinflammatory evaluation and molecular docking studies of N-((2-hydroxy-3-(2-(substitutedbenzylidene)hydrazine-1-carbonyl)naphthalen-1-yl)(3-nitrophenyl/3,4-dimethoxyphenyl)methyl)acetamide derivatives

**DOI:** 10.55730/1300-0527.3764

**Published:** 2025-04-18

**Authors:** Shikha KAMBOJ, Alka YADAV, Samridhi THAKRAL, Rekha TANWAR, Sunil KUMAR, Vikramjeet SINGH

**Affiliations:** 1 Department of Pharmaceutical Sciences, Faculty of Medical Sciences, Guru Jambheshwar University of Science and Technology, Haryana, India; 2 Guru Gobind Singh College of Pharmacy, Haryana, India

**Keywords:** Synthesis, antimicrobial, antiinflammatory, antioxidant, in silico studies

## Abstract

Hydrazone chemistry has become important and plays a key role in the development of organic compounds. Hydrazones can be useful pharmacophores for creating novel derivatives, owing to their broad range of activities. In this study, a series of N-((2-hydroxy-3-(2-(substitutedbenzylidene)hydrazine-1-carbonyl)naphthalen-1-yl)(3-nitro-phenyl/3,4-dimethoxyphenyl)methyl)acetamide derivatives were prepared, characterized by FTIR, ^1^H-NMR, ^13^C-NMR, and mass spectroscopy, and evaluated for their antimicrobial, antiinflammatory, and antioxidant activities along with in silico studies. The substituted derivatives were synthesized by the reaction of acetonitrile, chlorosulphonic acid, and substituted benzaldehyde with the hydrazones. The antimicrobial evaluation showed that compound 3i had more antimicrobial potential than the other tested molecules. Compound 3j had more antioxidant potential than the other synthesized compounds. Two compounds, 3f and 3h, had better antiinflammatory activity. The binding affinities of synthesized derivatives into the active sites of receptor proteins were characterized by utilizing the advanced docking program AutoDock Vina. In silico ADMET studies were performed using Molinspiration, pre-ADMET, and OSIRIS property explorer for the prediction of pharmacokinetic behavior of synthesized compounds.

## 1. Introduction

In recent years, hydrazones have become a versatile class of chemicals with numerous uses in organic synthesis and are easily obtained via condensation of hydrazines or hydrazides and carbonyl compounds [1]. They are crucial for the synthesis of heterocyclic compounds due to their biological and pharmacological characteristics [2]. Hydrazone chemistry has become important because of their key role in the development of organic compounds [3]. Despite substantial advances in antimicrobial therapy, infectious illnesses caused by bacteria and fungus continue to be a major global health concern due to the fast development of resistance to existing antibacterial and antifungal medications [4–5]. As a result, there is an urgent need to develop newer antimicrobial agents.

Infectious microorganisms like bacteria, viruses, or fungus frequently induce inflammation by entering the human body, dwelling in certain tissues, or participating in the circulation. Inflammation can also be caused by events such as tissue injury, cell death, malignancy, ischemia, and degeneration [6]. Inflammation is a complicated biological response of vascular tissues to harmful stimuli [7]. Nonsteroidal antiinflammatory drugs (NSAIDs) reduce the formation of prostaglandins, prostacyclins, and thromboxane derivatives and have significant cyclooxygenase inhibitory potential. COX-1 and COX-2, two cyclooxygenase isoforms, are well recognized The COX-2 enzyme system is associated with antiinflammatory properties, while the COX-1 enzyme system has both antiinflammatory and stomach irritant responses [8,9]. Antiinflammatory activity of a compound can be measured by assessing its abilitiy to prevent denaturation of a protein (e.g., egg albumin) via in vitro studies. Therefore, substances that can stop protein denaturation could be candidates for antiinflammatory medication development [10].

In recent years, free radical chemistry has become an active field of research. As a result of varied endogenous systems and exposure to distinct physiochemical environments, reactive oxygen species, reactive nitrogen species, and free radicals are generated by human body. Antioxidants have an important role in cellular and human body health [11,12]. Antioxidants are chemicals that slow down or prevent cellular damage by quenching or inhibiting free radical processes. Although antioxidant potential varies from species to species, they are present in all living things. Enzymatic and nonenzymatic types of antioxidants can be found in both the intracellular and extracellular environment [13,14]. Various reliable molecular modelling approaches have been used for drug development in pharmaceutical research. In particular, molecular docking algorithms have become more prevalent [15,16], each with relative strengths and weaknesses [15,17].

In continuation of our previous study [18] and reports related to computational studies and biological evaluation of potent molecules [19–21], the present study aimed to synthesize N-((2-hydroxy-3-(2-(substitutedbenzylidene) hydrazine-1-carbonyl)naphthalen-1-yl)(3-nitrophenyl/3,4-dimethoxyphenyl)methyl) acetamide derivatives, followed by their characterization and biological evaluation for antimicrobial, antioxidant, and antiinflammatory potential along with in silico activities.

## 2. Materials and methods

### 2.1. General procedure for the synthesis of N-((2-hydroxy-3-(2-(substitutedbenzylidene)hydrazine-1-carbonyl)naphthalen-1-yl)(3-nitrophenyl/3,4-dimethoxyphenyl)methyl)acetamide derivatives (2a–j, 3a–j, and 1a–j)

Synthesis of 3-hydroxy-N’-(substituted benzylidene)-2-naphthohydrazide (1a–j) was performed via a 3–4 h reflux reaction of 5 mmol 3-hydroxy-2-naphthoic acid hydrazide and 5 mmol substituted benzaldehyde in 30 mL of ethanol, as described in our previous study [18]. The solution of 3-hydroxy-N’-(substituted benzylidene)-2-naphthohydrazide (0.7 mmol) (1) and meta-NO_2_ benzaldehyde/3,4-dimethoxy benzaldehyde (0.7 mmol) in 15 mL of acetonitrile was dissolved using magnetic stirrer at room temperature and 0.7 mmol chlorosulphonic acid was added after the completion of the stirring. A clean reaction took place that was completed in 2–3 h and it was assessed by thin-layer chromatography (TLC) analysis. After pouring the reaction mixture into ice water, precipitates were obtained that were dried up at room temperature [22,23].

### 2.2. Physicochemical and spectral characterization

The physicochemical and spectral characterization of synthesized compounds are presented in Table 1. Graphical representation of all the spectral data is included in the supporting information.

### 2.3. Biological evaluation

#### 2.3.1 Antimicrobial activity

The microorganism suspension was created by assimilation of 1 mL of fresh culture to 100 mL of sterile saline solution. A 1000 μg/mL test solution was made and diluted using DMSO to 100 μg/mL. Nutrient broth and Sabouraud dextrose broth media were prepared, and 1 mL of medium was poured into a test tube and autoclaved. A 1 mL aliquot of the test compounds was added to the first test tube, and the next five test tubes were serially diluted to create dilutions of 50, 25, 12.50, 6.25, and 3.12 μg/mL. A 0.1 mL microbe suspension was added to each test tube to inoculate them. The bacterial strains were incubated for 24 h at 37 °C, while the fungal strains, *Rhizopus oryzae* and *Candida albicans*, were incubated for 5 days and 48 h, respectively. The lowest amounts that prevented microbial growth were then used to estimate the minimum inhibitory concentration (MIC) [18,24,25].

#### 2.3.2 Antiinflammatory activity

Antiinflammatory activity of the synthesized compounds was tested according to methods described in previous studies [18,10,26]. Solutions were prepared containing the synthesized compounds at varying concentrations in DMSO (500, 250, 125, 62.5, and 31.25 μg/mL). In each test tube, 1 mL of sample, 1.4 mL of freshly prepared phosphate buffer (pH 6.4), and 0.1 mL of egg albumin were added to test antiinflammatory activity. After incubation for 15 min at 37 ± 2 °C in a biochemical oxygen demand chamber, followed by heating at 70 °C for 5 min, the absorbance was measured using UV spectroscopy at 660 nm.

#### 2.3.3 Antioxidant activity

Antioxidant activity was evaluated using the 2,2-Diphenyl-1-picrylhydrazyl (DPPH) assay. Solutions containing the synthesized compounds were prepared in DMSO at different concentrations (500, 250, 125, 62.5, and 31.25 μg/mL). After adding 1 mL of DPPH solution to each test tube containing 1 mL of sample, the test tubes were all kept in a dark chamber for 30 min, during which the purple color turned yellow. The absorbance was then measured using UV spectroscopy at a wavelength of 517 nm. Readings were compared to a control of 1 mL of DMSO and 1 mL of DPPH, and DMSO was used as a blank [18,27].

### 2.4. Molecular docking

The advanced docking program AutoDock Vina was utilized for the prediction of binding parameters of N-((2-hydroxy-3-(2-(substitutedbenzylidene)hydrazine-1-carbonyl)naphthalen-1-yl)(3-nitrophenyl/3,4-dimethoxy phenyl)methyl)acetamide derivatives into the active sites of target proteins [24,25,28]. The 2D structures of ligands were outlined as stated in previous study [18].

The crystal structures of PDB 5H67 [29], PDB 5TZ1 [30], PDB 4Z69 [31–32], and PDB 1DXO [33] were prepared in pdbqt format and grid box was created using AutoDock tools as described in our previous study [18]. The results were visualized using Discovery Studio Visualizer and PyMol [34,35].

### 2.5. Statistical analysis

In vitro antiinflammatory and antioxidant readings were expressed as mean ± standard error of the mean (SEM). Dunnet’s post hoc multiple comparison test was used, followed by ANOVA. GraphPad Instat software was used to estimate p-values by performing a 1-way comparison test [18,36,37].

### 2.6. Pharmacokinetic properties

The online tool kits Molinspiration^1^, PreADMET^2^, and OSIRIS property explorer were used to compute drug-like features [36–38].

## 3. Results

### 3.1. Chemistry

The synthesis of the N-((2-hydroxy-3-(2-(substitutedbenzylidene)hydrazine-1-carbonyl)naphthalen-1-yl)(3-nitrophenyl)methyl) acetamide (2a–j) and N-((3,4-dimethoxyphenyl)(2-hydroxy-3-(2-(substituted benzylidene)hydrazine-1-carbonyl)naphthalen-1-yl)methyl)acetamide derivatives (3a–j) is shown in Scheme.

To synthesize the target compounds different 3-hydroxy-2-naphthoic acid hydrazones were reacted with metanitro benzaldehyde/3,4 dimethoxy benzaldehyde in the presence of acetonitrile and chlorosulfonic acid by refluxing for 2–3 h. The completion of reaction was confirmed using TLC analysis and the precipitates collected were filtered and dried. The purity of the synthesized compounds was evaluated by TLC analysis using silica gel G plates, along with Fourier transform-infrared (FT-IR), ^1^H nuclear magnetic resonance (NMR), ^13^CNMR, and mass spectra to confirm the successful synthesis of the desired compounds. The delineation of particular distinctive IR band positions suggested the synthesis of intended compounds with various substituents (Table 2).

The IR vibrations 3208–3300 cm^−1^ (OH stretch), 3410–3436 cm^−1^ (NH stretch), 3053–3077 cm^−1^ (C–H aromatic stretch), 2835–2962 cm^−1^ (C–H aliphatic stretch), 1590–1643 cm^−1^ (C=N stretch), 1441–1464 cm^−1^ (C=C aromatic stretch), and 1618–1652 cm^−1^ (C=O stretch) were observed for the synthesized derivatives. The presence of other functional groups, i.e. 1525–1549 cm^−1^ (NO_2_ asymmetric stretch), 1350–1397 cm^−1^ (NO_2_ symmetric stretch), and 1041–1100 cm^−1^ (O–CH_3_ stretch) were also observed.

The ^1^HNMR spectra of the synthesized compounds showed the presence of peaks at δ 11.36–12.30 ppm (N–H proton), δ 11.22–12.50 ppm (s) (OH group), δ 7.08–9.57 ppm (s) (H; C_h_ of ring: A), δ 7.25–8.69 ppm (d) (2H; C_d_ and C_g_ of ring: A), δ 7.34–8.10 ppm (t) (2H; C_e_ and C_f_ of ring: A), δ 1.40–3.86 ppm (OCH_3_), 4.11 ppm (OCH_2_), and 1.89–3.88 ppm (CH_3_ group). This confirmed the structure of synthesized compounds. The synthesized compounds had various peaks in the ^13^CNMR spectra around 160–185 ppm (C=O peak), 111–151.54 ppm (C=C atoms), 148–152.97 ppm (C=N group), 154 ppm (C–OH), 55–57 ppm (OCH_3_ group), and 35 ppm (CH_2_ group). The synthesized compounds had mass fragments that confirmed to their respective masses. Physicochemical properties of synthesized analogues are described in Table 3.

### 3.2. Biological evaluation

#### 3.2.1. In vitro antimicrobial activity

The antimicrobial activity of the synthesized compounds was estimated against gram-positive bacteria (*Bacillus subtilis*, *Staphylococcus aureus*), gram-negative bacteria (*Escherichia coli*, *Pseudomonas aeruginosa*), and fungal strains (*C*. *albicans*, *R*. *oryzae*) using a serial dilution procedure. Antimicrobial activity was obtained for each synthesized compound in terms of MIC values (Table 4).

Compounds 3i and 3j were found to be most effective against the *E*. *coli* with MIC values 0.011 and 0.021 μM, respectively, whereas 2j, 3b, and 3c had reduced activity against *E*. *coli* with MIC values of 0.044, 0.049, and 0.045 μM, respectively. Compounds 3i and 2c were effective against *P*. *aeruginosa*, with both showing MIC values of 0.006 μM. Compounds 2i, 3b, and 3h had reduced antibacterial potential against *P*. *aeruginosa* with MIC values of 0.046, 0.049, and 0.046 μM, respectively.

The growth of *S*. *aureus* was inhibited by compounds 2f and 3i, with the lowest MIC values of 0.011 and 0.006 μM, respectively. The least inhibitory compounds were 3c and 3g with MIC values of 0.045 and 0.044 μM, respectively. Compounds 2e, 3a, and 3d had MIC values of 0.006 μM and were the most active agents against *B*. *subtilis*. Compound 3i also had good antibacterial activity against *B*. *subtilis* with a MIC value of 0.011 μM. The least effective compounds against *B*. *subtilis* were compounds 2a, 2b, 2h, and 3b with MIC values of 0.024 and 0.025 μM. Amid all the synthesized analogues, compounds 2c and 3i had the highest antibacterial potential at a concentration of 0.006 μM against all bacterial strains.

With regards to antifungal activity, compounds 3b, 3c, 3h, and 3i were the most potent antifungal agents against *C*. *albicans* with MIC values of 0.012 and 0.011 μM (3c, 3h and 3i). The compounds 2i and 2j had MIC values of 0.092 and 0.044 μM, respectively, and they were the least effective against *C*. *albicans*. In the case of *R*. *oryzae*, growth was inhibited by compounds 2d, 3i, 3a, and 3d with MIC values of 0.006 (2d and 3i), and 0.011 μM (3a and 3d). Compounds 2b and 3j were the least active, with MIC values of 0.025 and 0.037 μM, respectively. Compounds 2d and 3i were the most potent antifungal agents, both with MIC values of 0.006 μM. Among tested compounds, 3i was the only compound that was active against all the tested bacteria and fungi.

#### 3.2.2. Antiinflammatory activity

The antiinflammatory activity of the N-((2-hydroxy-3-(2-(substitutedbenzylidene)hydrazine-1-carbonyl) naphthalen-1-yl)(3-nitrophenyl/3,4-dimethoxyphenyl)methyl)acetamide derivatives (2a–j and 3a–j) was evaluated using the egg albumin assay method. Ibuprofen was used as the reference drug, and all synthesized analogues were compared to it as described in Table 5. Compounds 2j, 3h, and 3f were the most efficacious compounds compared to the reference drug, with IC_50_ values of 0.09 and 0.05 μM, respectively. Compounds 2b, 3g, and 3i had the least inhibitory potential with IC_50_ values of 1.62, 1.45, and 0.96 μM, respectively.

#### 3.2.3. Antioxidant activity

N-((2-Hydroxy-3-(2-(substitutedbenzylidene)hydrazine-1-carbonyl)naphthalen-1-yl)(3-nitro-phenyl/3,4-di-methoxyphenyl)methyl)acetamide derivatives (2a–j and 3a–j) were tested for their antioxidant activity via the DPPH assay. Each process was repeated 3 times, and the average data were reported (Table 6). Compounds 3e, 3g, and 3j were the most potential antioxidant agent with IC_50_ values of 0.38, 0.22, and 0.20 μM respectively. Compounds 2f, 3c, 3d, and 3h had reduced activity as an antioxidant agent with IC_50_ values of 1.67, 1.45, 1.40, and 1.58 μM, respectively.

### 3.3. Molecular docking

Molecular docking utilizes computational algorithms to predict and visualize the binding of ligands with the receptor protein and estimate the binding energy. Three-dimensional views and various docking confirmations of the derived compounds help to understand into the pockets of the receptor proteins. Antibacterial results showed that all synthesized derivatives had binding energy varying from −7.3 to −9.4 kcal/mol (Table 7).

**Figure 1:**
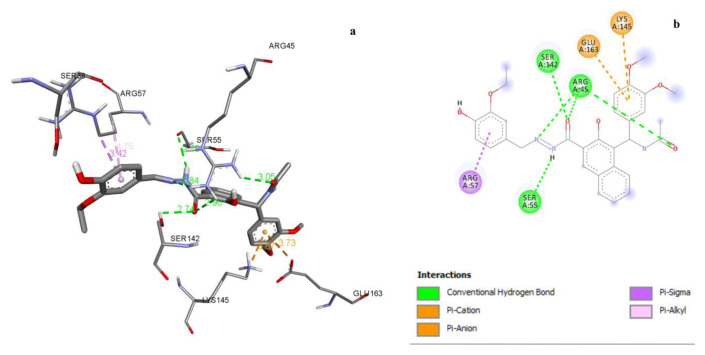
a) 3D docking view of compound 3i with PDB 5H67, b) 2D docking view of compound 3i with PDB 5H67.

Compound 3i established 5 H-bonds, electrostatic interactions, and pisigma interactions with the active pocket of PDB 5H67 (Figure 1, Table 8). The least active compound, 3b, had 3 H-bonds, 1 pi-cation, 1 pi-anion, and 1 amide-pi-stacked interaction with target residues.

**Figure 2:**
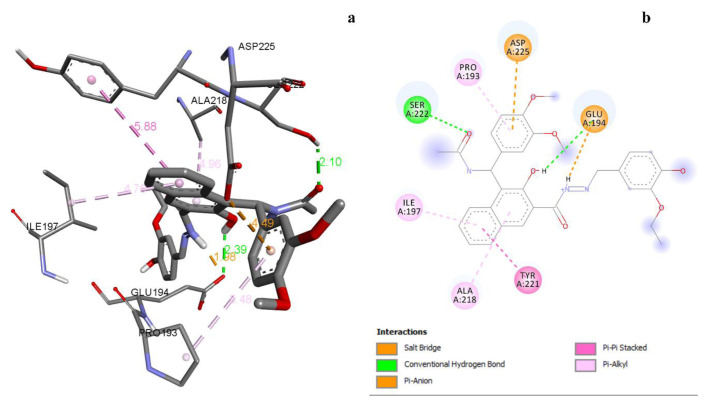
a) 3D docking view of compound 3i with PDB 5TZ1, b) 2D docking view of compound 3i with PDB 5TZ1.

All synthesized compounds showed binding energy ranging from −7.7 to −10.3 kcal/mol against the fungal protein (Table 7). Compound 3i had H-bond, electrostatic, and hydrophobic interactions with the active site of PDB 5TZ1 (Figure 2). The second most active compound, 3h, had H-bonds and hydrophobic interactions with amino acid residues (Table 9). Two hydrogen bond interactions, one pi-cation, 2 charge-charge and one pi-alkyl interactions were observed for compound 2i (the least active) with GluA:165, ThrA:172, PheA:198, GluA:194, and IleA:197 amino acid residues.

**Figure 3:**
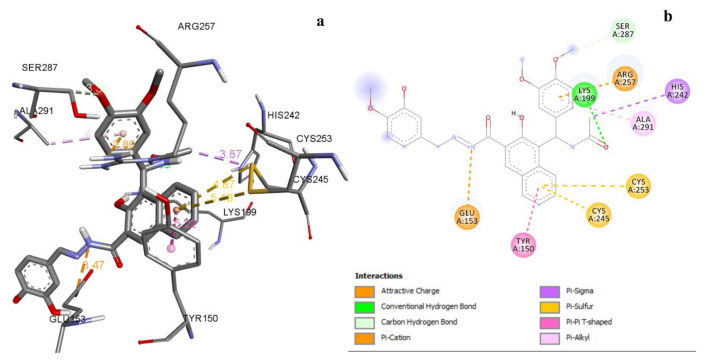
a) 3D docking view of compound 3h with PDB 4Z69, b) 2D docking view of compound 3h with PDB 4Z69.

Within the PDB 4Z69 active site, all compounds had binding energy in the range of −7.9 to −10.9 kcal/mol (Table 7). Compound 3h had electrostatic, hydrophobic, and H-bond interactions with target amino acid residues of PDB 4Z69 (Figure 3, Table 10). The least active compound, 2b, had H-bond interactions with GluA:188 and SerA:287; pi-alkyl interactions with LeuA:260, ValA:241, LeuA:238, and ArgA:218; and attractive charges with GluA:153 and GluA:292 amino acid residues.

**Figure 4:**
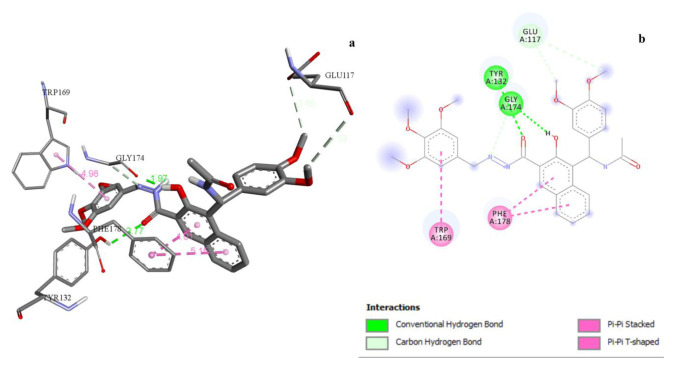
a) 3D docking view of compound 3j with PDB 1DXO, b) 2D docking view of compound 3j with PDB 1DXO.

Regarding the antioxidant activity, all derived compounds had binding energy ranging between −7.0 and −9.6 kcal/mol (Table 7). The most active compound, 3j, had hydrophobic and H-bond interactions with the active site of PDB 1DXO (Figure 4, Table 11). The least active compound 3c had 1 H-bond interaction and 2 pi-pi stacked interactions with HisA:257 and PheA:228 amino acid residues.

### 3.4. Calculation of pharmacokinetic properties

#### 3.4.1. Calculation of drug likeness parameters

Molinspiration and OSIRIS property tools were used for the evaluation of drug-like attributes. All the standard values have been mentioned in our previous study [18].

Some of the synthesized compounds had log p-values more than 5. The solubility values of all the derivatives were greater than −4. Most compounds had good topological polar surface area (TPSA) values and only the compound 3j had 11 rotatable bonds. Additionally, all compounds had optimum values as shown in Table 12.

#### 3.4.2. ADMET study

The pharmacokinetic parameters were calculated using the Pre-ADMET web server, as shown in Tables 13 and 14.

#### 3.4.3. Bioactivity and toxicity risk

The Molinspiration web service showed the bioactivity and toxicity of synthesized compounds and OSIRIS property explorer was used to anticipate mutagenicity, unwanted effects, and reproductive outcomes (Table 15).

## 4. Discussion

### Structure activity relationship (SAR)

The antimicrobial activity results indicated that compound 3i, which contains a 3-OC_2_H_5_-4-OH substituted benzylidene ring and a 3,4-diOCH_3_ substituted phenyl ring, exhibited the highest antimicrobial potential against all bacterial and fungal compared with the other compounds. The significance of the 3-OC_2_H_5_-4-OH substitution on the benzylidene ring in enhancing antimicrobial potential is supported by Narang et al. [39].

The antiinflammatory activity assays showed that compound 3h, bearing a di-OCH_3_ substituted phenyl ring and 3-OH-4-OCH_3_ substituted benzylidene, had the most antiinflammatory potential compared to compound 2h (metanitro substituted phenyl ring and 3-OH-4-OCH_3_ substituted benzylidene ring).

Compound 2g, bearing both electron-withdrawing groups (3,4-diCl substituted benzylidene ring and metanitro substituted phenyl ring), was superior to compound 3g possessing electron-withdrawing (3,4-diCl substituted benzylidene ring) and electron-donating group (di-OCH_3_ substituted phenyl ring). This is in agreement with Noriega et al. [40]. Nitrated derivatives of currently available drugs can be created to boost activity or lessen adverse consequences, as is the case with nitrated NSAIDs.

Antioxidant activity predicted that compound 3j, with a 3,4,5-tri-OCH_3_ substituted phenyl ring, had the more antioxidant potential than compounds 3c and 3d that had 2,5-diOCH_3_ and 3,4-di-OCH_3_ substituted phenyl rings, respectively. Lee et al. [41], Sudha et al. [42], and Selvam et al. [43] reported that compound bearing electron-donating groups had maximum inhibitory potential [41–43].

Compound 3g, with 3,4-diCl substituted benzylidene and di-OCH_3_ substituted phenyl rings, and 3e, with 2,3-diCl substituted benzylidene and di-OCH_3_ substituted phenyl rings had both electron-withdrawing and electron-donating groups that enhanced antioxidant activity. This was correlated with compounds 2g and 2e that had electron-withdrawing groups. The role of electron-withdrawing groups in diminishing the antioxidant potential is explained by Alam et al. [44].

The synthesis of N-((2-hydroxy-3-(2-(substituted benzylidene)hydrazine-1-carbonyl)naphthalen-1-yl)(3-nitrophenyl/3,4-dimethoxyphenyl)methyl)acetamide derivatives (2a–j and 3a–j) was accomplished with a multicomponent reaction. The molecular structures of compounds were supported by FT-IR, ^1^HNMR, ^13^CNMR, and MS spectra. The series of N-((2-hydroxy-3-(2-(substituted benzylidene)hydrazine-1-carbonyl)naphthalen-1-yl)(3-nitrophenyl/3,4-dimethoxyphenyl)methyl) acetamide derivatives was examined for their biological potential and in silico studies examined their binding activity in more detail. The results of antimicrobial activity showed that compound 3j had the highest potential against the tested bacteria and fungi. Compounds 3f and 3h were the most active antiinflammatory agents, and compound 3j showed the highest antioxidant potential. Molecular docking investigations showed strong binding affinities of the synthesized compounds and existence of various hydrophobic, electrostatic, and hydrogen bonding interactions. Furthermore, in silico ADMET screening results showed that the synthesized compounds had favorable properties.

## Supplementary Information



## Figures and Tables

**Scheme:**
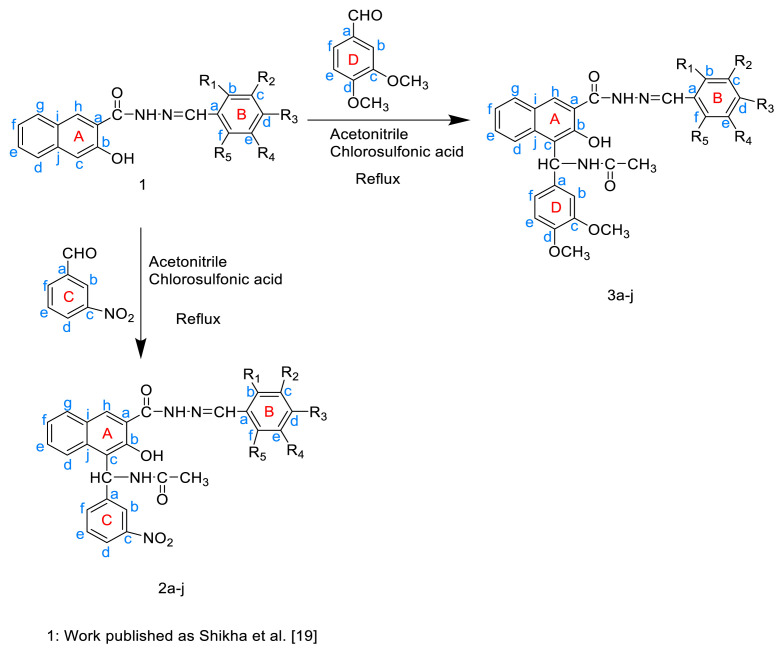
General scheme for the synthesis of N-((2-hydroxy-3-(2-(substituted benzylidene)hydrazine-1-carbonyl)naphthalen-1-yl)(3-nitrophenyl/3,4-dimethoxy-phenyl)methyl)acetamide derivatives.

**Table 1:** Analytical data of the synthesized compounds.

Comp.	Analytical data
	a- IR (cm^−1^); b- ^1^HNMR (DMSO d_6_ δppm); c- ^13^CNMR (DMSO d_6_ δppm); d-Mass
**2a**	**a-** 3417 (NH- str. of  ), 3330 (OH str. of ring: A), 3077 (CH- aromatic str.), 2931 (CH- aliphatic str.), 1638 (C=O str. of 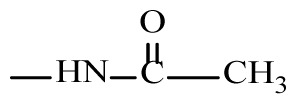 ), 1618 (C=O str. of  ), 1597 (C=N stretching of ring: B), 1526 (NO_2_: Asymmetric str.), 1450 (C=C aromatic str.), 1397 ( NO_2_: Symmetric str.)
**2b**	**a-** 3417 (NH- str. of  ), 3242 (OH str. of ring: A), 3074 (CH- aromatic str.), 2928 (CH aliphatic stretching), 1639 (C=O str. of 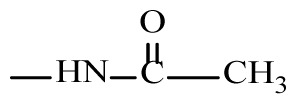 ), 1617 (C=O str. of  ), 1526 (NO_2_: Symmetric str.), 1450 (C=C- aromatic stretching), 1350 (NO_2_: Asymmetric str.)
**2c**	**a-** 3417 (NH- str. of  ), 3243 (OH str. of ring: A), 2843(CH- aliphatic str.), 1638 (C=O str. of 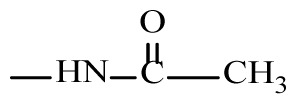 ), 1618 (C=O stretching of  ), 1526 (NO_2_: Symmetric str.), 1450 (C=C- aromatic str.), 1350 (NO_2_: Asymmetric str.), 1071 (OCH_3_ str. of C_b_ of ring: B), 1043 (OCH_3_ str. of C_e_ of ring: B); **b-** 12.16 (s, 1H, NH), 12.05 (s, 1H, OH of C_b_ of ring: A), 11.22 (s, 1H of secondary amine), 8.78 (s, 1H of ring: B), 8.59 (s, 1H of CH-NH-), 8.45–8.47 (d, 1H of C_d_ of ring: C, J 8 Hz), 8.28–8.30 (d, 1H of C_f_ of ring: C, J 8 Hz), 8.19–8.21 (d, 2H of C_d_ and C_g_ of ring: A, J 8 Hz), 7.91–7.95 (t, 1H of C_e_ of ring: C, J 8 Hz), 7.77–7.81 (t, 2H of C_e_ and C_f_ of ring: A, J 8 Hz), 7.32–7.34 (m, 3H of ring: B), 3.88 (s, 3H of methyl group), 3.84 (s, 3H of OCH_3_ of C_b_ of ring: B), 3.78 (s, 3H of OCH_3_ of C_e_ of ring: B); **c-**164.73 (C=O), 164.28 (C=O of methyl acetamide), 154.98 (C-OH), 154.18 (C_b_ of ring: B), 153.78 (C_e_ of ring: B), 152.97 (CH of ring: B), 148.74 (C-NO_2_), 148.27 (C_j_ of ring: A), 146.48 (C_i_ of ring: A), 144.53 (C_a_ of ring: A), 136.38 (C_e_ and C_f_ of ring: A), 136.54 (C_h_ of ring: A),133.89(CH of methyl acetamide),136.34 (C_a_ of ring: B), 133.26 (C_a_ of nitro phenyl ring:), 131.06 (C_f_ of nitro phenyl ring), 131.01 (C_e_ and C_h_ of ring: A), 130.99 (C_d_ of ring: A), 130.34 (C_f_ of ring: B), 129.17 (C_c_ of ring: B), 129.10 (C_d_ of ring: B), 128.77 (C_b_ of ring: C), 127.31 (C_e_ of ring: C), 127.18 (C_d_ of ring: C), 56.78 (OCH_3_ of C_b_ of ring: B), 55.91 (OCH_3_ of C_c_ of ring: B), 22.96 (CH_3_ of methyl acetamide) d- m/z(M+1 observed): 543.18; m/z (M actual): 543
**2d**	**a-** 3417 (NH- str. of  ), 3242 (OH str. of ring: A), 2938 (CH- aliphatic str.), 1637 (C=O str. of 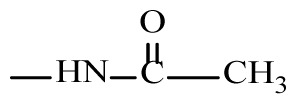 ), 1618 (C=O str. of  ), 1525 (NO_2_: Symmetric str.), 1450 (C=C- aromatic str.), 1071 (OCH_3_ str. of ring: B)
**2e**	**a-** 3416 (NH- str. of  ), 3238 (OH str. of ring: A), 2914 (CH- aliphatic str.), 1642 (C=O str. of  ), 1625 (C=O str. of 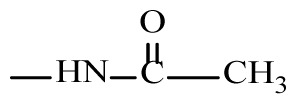 ), 1549 (NO_2_: Symmetric str.), 1454 (C=C- aromatic str.), 1357 (NO_2_: Asymmetric str.), 625 (C-Cl str. of C_b_ of ring: B), 742 (C-Cl str. of C_c_ of ring: B)
**2f**	**a-** 3436 (NH- str. of 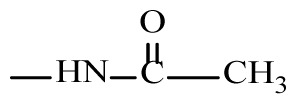 ), 3322 (NH- str. of  ), 3265 (OH str. of ring: A), 3057 (CH- aromatic str.), 1647 (C=O str. of 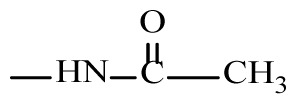 ), 1584 (C=N str.), 1530 (NO_2_: Symmetric str.), 1443 (C=C- aromatic str.), 758 (C-Cl str. of C_b_ of ring: B), 740 (C-Cl str. of C_f_ of ring: B); **b-** 12.30 (s, 1H, NH), 12.23 (s, 1H, OH of C_b_ of ring: A), 11.16 (s, 1H of secondary amine), 8.75–8.78 (d, 1H of C_f_ ring: C, J 8 Hz), 8.71–8.73 (d, 1H of C_d_ of ring: C, J 8 Hz), 8.45 (s, 1H of ring: B), 8.67–8.69 (d, 2H of C_d_ and C_g_ of ring: A, J 8 Hz), 8.62 (s, 1H of C_b_ of ring: C), 8.23–8.25 (d, 2H of C_c_ and C_e_ of ring: B, J 8 Hz), 8.06–8.10 (t, 2H of C_e_ and C_f_ of ring: A, J 8 Hz), 7.77–7.82 (t, 1H of C_e_ of ring: C, J 8 Hz), 7.58–7.62 (t, H of C_d_ of ring: B), 7.33 (s, 1H of C_h_ of ring: A), 2.04 (s, 3H of methyl); **c-** 170.29 (C=O), 164.61 (C=O of methyl acetamide), 154.47 (C-OH), 148.77 (C-NO_2_), 148.28 (CH of ring: B), 148.11 (C_a_ of ring: C), 146.13 (C_f_ of ring: C), 144.29 (C_b_ of ring: B), 136.38 (C_b_ of ring: B),136.14 (C_j_ of ring: A), 134.18 (C_i_ of ring: A), 134.18 (C_a_ of ring: A),133.28 (C_h_ of ring: A), 131.82 (C_e_ of ring: A) 131.07 (C_f_ of ring: A), 130.98 (C_g_ of ring: A), 130.78 (C_d_ of ring: A), 130.58 (C_a_ of ring: B), 130.19 (C_e_ of ring: B), 129.60 (C_d_ of ring: B), 129.15 (C_b_ of ring: C), 128.76 (C_c_ of ring: A), 127.25 (C_e_ of ring: C), 126.33 (C_e_ of ring: C), 125.72 (C_d_ of ring: C), 124.30 (C_c_ of ring: B), 47.95 (CH of methyl acetamide), 22.96 (CH_3_ of methyl acetamide), 2.04 (CH_3_ of methyl acetamide)
**2j**	a- 3436 (NH- str. of 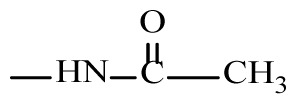 ), 3246 (OH str. of ring: A), 3075 (CH- aromatic str.), 2893 (OCH_3_ str.), 1636 (C=O str. of 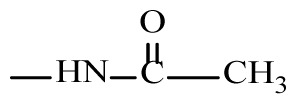 ), 1597 (C=N str.), 1524 (NO_2_: Symmetric str.), 1450 (C=C- aromatic str.), 1071 (OCH_2_CH_3_ str.); b- 12.16 (s, 1H, NH), 11.22 (s, 1H, OH of C_b_ of ring: A), 11.95 (s, 1H of secondary amine), 8.59 (s, 1H of ring: B), 8.46 (s, 1H of CH of methyl acetamide), 8.39 (s, 1H of ring: C), 8.28–8.30 (d, 1H of C_d_ of ring: C, J 8 Hz), 8.19–8.21 (d, 2H of C_d_ and C_g_ of ring: A, J 8 Hz), 7.49–7.54 (t, 1H of C_e_ of ring: C), 7.35–7.39 (t, 2H of C_f_ and C_g_ of ring: B, J 8 Hz), 7.75–7.95 (m, 2H of ring: B), 7.08 (s, 1H of C_h_ of ring: A), 3.86 (s, 6H of methoxy group of C_c_ and C_e_ of ring: B), 3.73 (s, 3H of methoxy group of C_e_ of ring: B), 2.51 (s, 3H of methyl); **c-**166.39 (C=O), 164.27 (C=O of methyl acetamide), 154.35 (C-OH), 154.20 (C_c_ of ring: B), 148.74 (C_d_ of ring: B), 145.47(C_e_ of ring: B), 136.56 (C_e_ of ring: C), 136.44 (CH of ring: B), 136.34 (C_i_ and C_j_ of ring: A), 133.90 (C_a_ of ring: C), 131.84 (C_f_ of ring: C), 131.06 (C_e_ and C_f_ of ring: A), 131.00 (C_d_ of ring: C), 129.25 (C_h_ of ring: A), 129.16 (C_d_ of ring: A), 128.84 (C_a_ of ring: B), 128.75 (C_f_ of ring: B), 127.31 (C_b_ of ring: B), 126.28 (C_c_ of ring: A), 124.89 (CH of methyl acetamide), 121.62 (C_b_ of ring: C), 121.13 (C_h_ of ring: A), 118. 97 (C_e_ of ring: C), 111.04 (C_a_ of ring: A), 56.48 (OCH_3_ of ring: B)
**3a**	**a-** 3416 (NH- str. of  ), 2926 (CH- aliphatic str.), 1638 (C=C- str. of 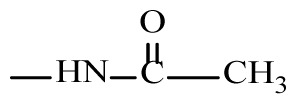 ), 1619 (C=O str. of  ), 1597 (C=N str.), 1350 (NO_2_: Asymmetric str), 1450 (C=C- aromatic str.), 1071 (OCH_3_ str.of C_c_ of ring: D), 1023 (OCH_3_ str. of C_d_ of ring: D)
**3b**	**a-** 3416 (NH- str. of  ), 2925 (CH- aliphatic str.), 1639 (C=O str. of  ), 1618 (C=O str. of 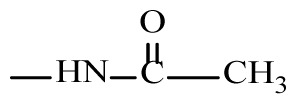 ), 1450 (C=C aromatic str.)
**3c**	**a-** 3415 NH- str. of  ), 2835 (CH- aliphatic str.), 1639(C=O str. of  ), 1618 (C=O str. of 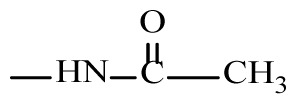 ), 1464 (C=C- aromatic str.), 1076 (OCH_3_ str. of C_c_ of ring: D), 1043 (OCH_3_ str. of ring: B)
**3d**	**a-** 3410 (NH- str. of  ), 3243 (OH str. of ring: A), 3053 (CH- aromatic str.), 2935 (CH- aliphatic str.), 1612 (C=O str. of  ), 1601 (C=O str. of 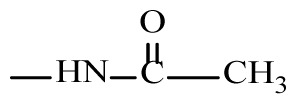 ), 1451 (C=C- aromatic str.), 1641 (C=N str.), 1023 (OCH_3_ str. of ring: D)
**3e**	**a-** 3418 (NH- str. of  ), 3072 (CH- aromatic str.), 1643 (C=O str. of  ), 1626 (C=O str. of 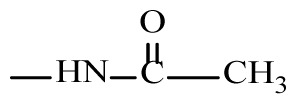 ), 1599 (C=N str.), 1454 (C=C- aromatic str.), 1074 (OCH_3_ of C_c_ of ring: D), 1042 (OCH_3_ str. of C_d_ of ring: D), 742 (C-Cl str. of C_b_ of ring: B), 708 (C-Cl str. of C_c_ of ring: B)
**3f**	**a-** 3416 (NH- str. of  ), 3208 (OH str. of ring: A), 3056 (CH- aromatic str.), 1652 (C=O str. of  ), 1623 (C=O str. of 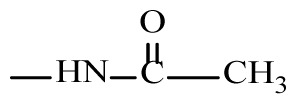 ), 1595 (C=N str.), 1464 (C=C- aromatic str.), 1089 (OCH_3_ str. of ring: D), 742 (C-Cl str. of C_b_ of ring: B), 775 (C-Cl str. of C_f_ of ring: B)
**3g**	**a-** 3416 (NH- str. of  ), 3290 (OH str. of ring: A), 1638 (C=O str. of  ), 1625 (C=O str. of 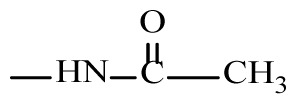 ), 1590 (C=N str.), 1443 (C=C- aromatic str.), 1097 (OCH_3_ str. of C_c_ of ring: D), 1029 (OCH_3_ str. of C_d_ of ring: D), 739 (C-Cl str. of C_c_ of ring: B), 601 (C-Cl str. of C_d_ of ring: B); **b-** 12.09 (s, 1H, NH), 11.22 (s, 1H, OH of C_b_ of ring: A), 8.66 (s, 1H of CH-NH-C=O-CH3), 8.44 (s, 1H of ring: B), 8.03 (s, 1H of C_h_ of ring: A), 8.00 (s, 1H of C_b_ of ring: B) 7.92–7.94 (d, 2H of C_d_ and C_g_ of ring: A, J 8 Hz), 7.50–7.52 (d, 1H of C_f_ of ring: B, J 8 Hz), 7.75–7.78 (s, 1H of C_e_ of ring: D), 7.34–7.39 (t, 2H of C_e_ and C_f_ of ring: A, J 8 Hz), 3.69 (s, 3H of methoxy group of C_d_ of ring: D), 3.85 (s, 3H of methoxy group of C_c_ of ring: D), 1.98 (s, 3H of methyl group); **c-** 164.13 (C=O), 154.64 (C-OH), 154.74 (C=O of methyl acetamide), 151.45 (C_c_ of ring: D), 149.97 (CH of ring: B), (C_d_ of ring: D), 149.59 (C_a_ of ring: D),149.29 (C_c_ of ring: B), 147.69 (C_d_ of ring: B), 136.30 (C_d_ of ring: D), 130.57 (C_j_), 129.12 (C_i_), 128.68 (C_a_ of ring: B), 127.43 (C_b_ of ring: B), 127.30 (C_f_ of ring: B), 127.25 (C_e_ of ring: B), 126.31 (C_e_ of ring: A),124.27(C_f_ of ring: A), 122.78 (C_a_ of ring: A), 122.62 (C_h_ of ring: A), 122.04 (C_c_ of ring: A), 120.76 (C_d_ of ring: A), 120.56 (C_g_ of ring: A), 116.07 (C_b_ of ring: D), 112.40 (C_e_ of ring: D), 111.06 (C_f_ of ring: D), 66.41 (CH of methyl acetamide), 56.02 (OCH_3_ str. of C_d_ of ring: D), 56.09 (OCH_3_ str. of C_c_ of ring: D), 55.98 (CH_3_ of methyl acetamide)
**3h**	**a-** 3416 (NH str. of  ), 3057 (CH aromatic str.), 2934 (CH aliphatic str.), 1650 (C=O str. of  ), 1619 (C=O str. of 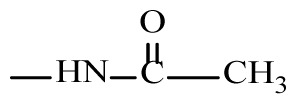 ), 1441 (C=C aromatic str.), 1068 (OCH_3_ str. of C_c_ and C_d_ of ring: D), 1023 (OCH_3_ str. of C_d_ of ring: B)
**3i**	**a-** 3412 (NH str. of  ), 3243 (OH str.), 3056 (CH aromatic str.), 2934 (CH aliphatic str.), 1643 (C=N str.), 1615 (C=O str. of  ), 1600 (C=O str. of 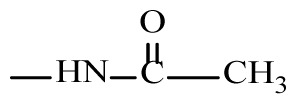 ), 1441 (C=C aromatic str.), 1023 (OCH_2_CH_3_ str. of C_c_ of ring: B); **b-** 11.90 (s, 1H, NH), 11.85 (s, 1H, OH of C_d_ of ring: B), 11.36 (s, 1H, OH of C_b_ of ring: A), 9.52 (s, 1H of CH of ring: B), 9.77 (s, 1H of ring: B), 8.46 (s, 1H of C_h_ of ring: A), 8.41 (s, 1H of C_b_ of ring: B) 8.34 (s, 1H of C_b_ of ring: D), 7.91–7.93 (d, 1H of C_f_ of ring: D, J 8 Hz), 7.76–7.78 (s, 2H of C_d_ and C_g_ of ring: A), 7.50–7.54 (t, 2H of C_e_ and C_f_ of ring: A, J 8Hz), 7.25 – 7.27 (d, 1H of C_e_ of ring: B, J 8 Hz), 7.13–7.15 (d, 1H of C_f_ of ring: B, J 8 Hz), 7.05–7.07 (d, 1H of C_e_ of ring: D, J 8 Hz), 4.11 (s, 2H of OCH_2_ of C_c_ of ring: B), 3.69 (s, 3H of methoxy group of C_d_ of ring: B), 3.33 (s, 3H of methoxy group of C_c_ of ring: B), 1.40 (s, 3H of CH_3_ of C_c_ of ring: B), 1.39 (s, 3H of CH_3_ of methyl acetamide); **c-** 191.85 (C=O), 164.13 (C=O of methyl acetamide),154.74 (C-OH), 154.64 (C_d_ of ring: B), 151.45 (C_c_ of ring: B), 149.59 (C_c_ of ring: D), 149.29 (C_d_ of ring: D), 147.69 (CH of ring: B), 136.30 (C_j_ of ring: A), 130.56 (C_i_ of ring: A), 129.12 (C_a_ of ring: A), 128.68 (C_h_ of ring: A), 127.30 (C_e_ and C_f_ of ring: A) 127.25 (C_f_ of ring: B), 126.31 (C_a_ of ring: D),124.27 (C_b_ of ring: D), 122.62 (C_e_ of ring: D), 122.77 (C_f_ of ring: D), 121.03 (C_g_ of ring: A), 120.72 (C_b_ of ring: B), 116.07(C_d_ of ring: A),112.03 (C_a_ of ring: B), 111.06 (C_c_ of ring: A), 108.9 (C_e_ of ring: B), 56.98 (OCH_2_CH_3_ of C_c_ of ring: B), 56.02 (OCH_3_ str.of C_d_ of ring: D), 56.09 (OCH_3_ str. of C_c_ of ring: D); **d-** m/z (M+1 observed): 558.22; m/z (M actual)
**3j**	**a-** 3415 (NH str. of  ), 3238 (OH str. of ring: A), 3056 (CH aromatic str.), 2962 (CH aliphatic str.), 1640 (C=O str. of  ), 1618 (C=O str. of 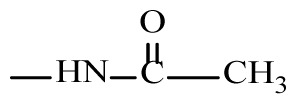 ), 1464 (C=C aromatic str.), 1072 (OCH_3_ str. of C_c_ and C_d_ of ring: D), 1025 (OCH_3_ str. of ring: B); **b-** 11.97 (s, 1H, NH), 11.90 (s, 1H, OH of C_b_ of ring: A),11.36 (s, 1H of CH-NH-C=O-),10.20 (s, 1H of ring: B), 8.40 (s, 1H of C_h_ of ring: A), 8.47 (s, 1H of C_a_ of ring: D), 7.75–7.78 (d, 1H of C_f_ of ring: D, J 12 Hz), 7.05–7.08 (d, 1H of C_e_ of ring: D, J 12 Hz), 7.25–7.27 (d, 2H of C_d_ and C_g_ of ring: A, J 8 Hz), 7.50 – 7.54 (t, 1H of C_f_ of ring: A, J 8 Hz), 7.89–7.93 (t, 1H of C_e_ of ring: A, J 8Hz), 7.32–7.39 (m, 2H of ring: B), 3.73 (s, 6H of OCH_3_ of C_c_ and C_e_ of ring: B), 3.54 (s, 3H of OCH_3_ of C_d_ and C_c_ of ring: D), 2.51(s, 3H of OCH_3_ of C_d_ of ring: B), 2.06 (s, 3H of CH_3_of methyl group); **c-** 166.34 (C=O), 164.13 (C=O of methyl acetamide), 154.65 (C-OH), 154.32 (C_c_ and C_e_ of ring: B), 151.43 (C_41_ of ring: B), 149.57 (C_d_ of ring: D), 149.29 (C_c_ of ring: D), 136.45 (CH of ring: B), 136.29 (C_i_ of ring: A), 130.90 (C_j_ of ring: A), 130.57 (C_g_ of ring: A), 129.27 (C_f_ of ring: A), 129.13 (C_e_ of ring: A), 128.86 (C_a_ of ring: D), 128.68 (C_a_ of ring: A),127.25 (C_d_ of ring: A), 127.21 (C_b_ of ring: B), 126.27 (C_f_ of ring: B), 124.29 (C_f_ of ring: A), 120.69 (C_b_ of ring: D), 118.97 (C_f_ of ring: D), 112.01 (C_e_ of ring: D), 111.04 (C_c_ of ring: A), 56.47 (OCH_3_ str. of C_c_ and C_e_ of ring: B), 56.08 (OCH_3_ str. of C_d_ of ring: B), 55.97 (OCH_3_ str. of C_c_ and C_d_ of ring: D); **d-** m/z(M+1 observed): 587.56; m/z (M actual): 587

**Table 2:** List of substituents for target compounds (2a–j and 3a–j).

Comp.	R_1_	R_2_	R_3_	R_4_	R_5_	X	Y
**2a**	H	NO_2_	H	H	H	NO_2_	H
**2b**	H	H	OH	H	H	NO_2_	H
**2c**	OCH_3_	H	H	OCH_3_	H	NO_2_	H
**2d**	H	OCH_3_	OCH_3_	H	H	NO_2_	H
**2e**	Cl	Cl	H	H	H	NO_2_	H
**2f**	Cl	H	H	H	Cl	NO_2_	H
**2g**	H	Cl	Cl	H	H	NO_2_	H
**2h**	H	OH	OCH_3_	H	H	NO_2_	H
**2i**	H	OC_2_H_5_	OH	H	H	NO_2_	H
**2j**	H	OCH_3_	OCH_3_	OCH_3_	H	NO_2_	H
**3a**	H	NO_2_	H	H	H	OCH_3_	OCH_3_
**3b**	H	H	OH	H	H	OCH_3_	OCH_3_
**3c**	OCH_3_	H	H	OCH_3_	H	OCH_3_	OCH_3_
**3d**	H	OCH_3_	OCH_3_	H	H	OCH_3_	OCH_3_
**3e**	Cl	Cl	H	H	H	OCH_3_	OCH_3_
**3f**	Cl	H	H	H	Cl	OCH_3_	OCH_3_
**3g**	H	Cl	Cl	H	H	OCH_3_	OCH_3_
**3h**	H	OH	OCH_3_	H	H	OCH_3_	OCH_3_
**3i**	H	OC_2_H_5_	OH	H	H	OCH_3_	OCH_3_
**3j**	H	OCH_3_	OCH_3_	OCH_3_	H	OCH_3_	OCH_3_

**Table 3:** Physicochemical properties of synthesized N-((2-hydroxy-3-(2-(substitutedbenzylidene)hydrazine-1-carbonyl)naphthalen-1-yl)(3-nitrophenyl/3,4-dimethoxy phenyl)methyl)acetamide derivatives (2a–j and 3a–j).

Comp.	Mol. wt.	Mol. Formula	Melting point (°C)	R_f_ value	% Yield
**2a**	527	C_27_H_21_N_5_O_7_	193–197	0.71^	73.50
**2b**	498	C_27_H_22_N_4_O_6_	198–202	0.49	30.04
**2c**	542	C_29_H_26_N_4_O_7_	180–184	0.66^	61.50
**2d**	542	C_29_H_26_N_4_O_7_	220–224	0.60*	59.59
**2e**	551	C_27_H_20_Cl_2_N_4_O_5_	218–222	0.68^	54.20
**2f**	551	C_27_H_20_Cl_2_N_4_O_5_	218–222	0.88^	73.76
**2g**	551	C_27_H_20_Cl_2_N_4_O_5_	220–224	0.55^	53.76
**2h**	528	C_28_H_24_N_4_O_7_	220–224	0.18	70.48
**2i**	542	C_29_H_26_N_4_O_7_	237–241	0.58	67.27
**2j**	572	C_30_H_28_N_4_O_8_	238–242	0.41^	67.02
**3a**	542	C_29_H_26_N_4_O_7_	118–122	0.44	83.90
**3b**	513	C2_9_H_27_N_3_O_6_	123–127	0.66	51.30
**3c**	557	C_31_H_31_N_3_O_7_	108–112	0.68*	60.60
**3d**	557	C_31_H_31_N_3_O_7_	178–182	0.57	68.90
**3e**	566	C_29_H_25_Cl_2_N_3_O_5_	178–182	0.96	67.30
**3f**	566	C_29_H_25_Cl_2_N_3_O_5_	198–202	0.62	66.58
**3g**	566	C_29_H_25_Cl_2_N_3_O_5_	180–184	0.63α	70.88
**3h**	543	C_30_H_29_N_3_O_7_	126–130	0.54	50.71
**3i**	557	C_31_H_31_N_3_O_7_	129–133	0.49β	58.08
**3j**	587	C_32_H_33_N_3_O_8_	127–131	0.52^d^	73.09

TLC mobile phase:

Benzene: Chloroform = (5:5)

* Toluene: Chloroform = (5:5)

^ Toluene: Chloroform = (1:9)

α Toluene: methanol= (9: 1)

β Benzene: Chloroform = (1:9)

d Methanol: Chloroform = (1:9)

**Table 4:** Antimicrobial activity of synthesized N-((2-hydroxy-3-(2-(substitutedbenzylidene)hydrazine-1-carbonyl)naphthalen-1-yl)(3-nitrophenyl/3,4-dimethoxy phenyl)methyl)acetamide derivatives (2a–j and 3a–j) (μM).

Comp.	*Escherichia coli*	*Pseudomonas aeruginosa*	*Staphylococcus aureus*	*Bacillus subtilis*	*Candida albicans*	*Rhizopus oryzae*
**2a**	0.024	0.024	0.024	0.024	0.024	0.024
**2b**	0.025	0.025	0.025	0.025	0.025	0.025
**2c**	0.023	0.006	0.023	0.023	0.023	0.023
**2d**	0.023	0.023	0.023	0.023	0.023	0.006
**2e**	0.023	0.023	0.023	0.011	0.023	0.023
**2f**	0.023	0.023	0.011	0.023	0.023	0.023
**2g**	0.023	0.023	0.023	0.023	0.023	0.023
**2h**	0.024	0.024	0.024	0.024	0.024	0.024
**2i**	0.023	0.046	0.023	0.023	0.092	0.023
**2j**	0.044	0.044	0.022	0.022	0.044	0.022
**3a**	0.023	0.023	0.023	0.011	0.023	0.011
**3b**	0.049	0.049	0.024	0.024	0.012	0.024
**3c**	0.045	0.045	0.045	0.022	0.011	0.022
**3d**	0.022	0.022	0.022	0.011	0.022	0.011
**3e**	0.022	0.022	0.022	0.022	0.022	0.022
**3f**	0.022	0.022	0.022	0.022	0.022	0.022
**3g**	0.022	0.022	0.044	0.022	0.022	0.022
**3h**	0.023	0.046	0.023	0.023	0.011	0.023
**3i**	0.011	0.006	0.006	0.006	0.011	0.006
**3j**	0.021	0.043	0.021	0.021	0.021	0.037
**Ciprofloxacin**	0.0037	0.0037	0.0037	0.0037	-	-
**Fluconazole**	-	-	-	-	0.0040	0.0040

**Table 5:** Antiinflammatory activity of synthesized N-((2-hydroxy-3-(2-(substitutedbenzylidene)hydrazine-1-carbonyl)naphthalen-1-yl)(3-nitrophenyl/3,4-dimethoxy phenyl)methyl)acetamide derivatives (2a–j and 3a–j) (mean percentage inhibition ± SEM).

Comp.	Conc.	Mean±SEM	IC_50_

**2a**	500	77.66±0.09**	0.36
	250	64.05±0.02**	
	125	58.06±0.02**	
	62.5	41.40±0.05**	
	31.25	37.82±0.15**	

**2b**	500	72.56±0.14**	1.62
	250	56.74±0.08**	
	125	42.36±0.01**	
	62.5	33.61±0.05**	
	31.25	25.77±0.05**	

**2c**	500	54.39±0.07**	0.32
	250	45.75±0.16**	
	125	33.10±0.03**	
	62.5	24.55±0.13**	
	31.25	20.02±0.01**	

**2d**	500	62.20±0.16**	0.46
	250	53.93±0.01**	
	125	45.27±0.15**	
	62.5	38.61±0.18**	
	31.25	33.45±0.14**	

**2e**	500	68.39±0.48**	0.2
	250	62.58±0.21**	
	125	53.81±0.16**	
	62.5	45.54±0.11**	
	31.25	38.38±0.13**	

**2f**	500	68.08±0.09**	0.22
	250	63.63±0.09**	
	125	52.30±0.09**	
	62.5	44.89±0.04**	
	31.25	37.66±0.02**	

**2g**	500	76.68±0.15**	0.29
	250	69.86±0.02**	
	125	59.29±0.21**	
	62.5	52.84±0.13**	
	31.25	45.87±0.14**	

**2h**	500	73.03±0.02**	0.15
	250	68.53±0.02**	
	125	56.55±0.20**	
	62.5	48.11±0.03**	
	31.25	37.11±0.09**	

**2i**	500	66.16±0.07**	0.47
	250	57.71±0.04**	
	125	48.27±0.13**	
	62.5	41.08±0.02**	
	31.25	34.10±0.03**	

**2j**	500	70.31±0.25**	0.09
	250	64.63±0.06**	
	125	59.68±0.16**	
	62.5	48.36±0.18**	
	31.25	38.64±0.01**	

**3a**	500	62.27±0.08**	0.78
	250	51.06±0.02**	
	125	39.64±0.04**	
	62.5	30.95±0.02**	
	31.25	22.92±0.00**	

**3b**	500	73.50±0.43**	0.17
	250	67.61±0.03**	
	125	55.14±0.01**	
	62.5	47.08±0.03**	
	31.25	36.10±0.04**	

**3c**	500	77.39±0.21**	0.23
	250	68.95±0.01**	
	125	55.82±0.01**	
	62.5	46.56±0.33**	
	31.25	35.33±0.00**	

**3d**	500	68.69±0.21**	0.31
	250	61.12±0.21**	
	125	49.97±0.12**	
	62.5	43.25±0.31**	
	31.25	36.06±0.07**	

**3e**	500	68.04±0.03**	0.25
	250	61.74±0.01**	
	125	51.38±0.04**	
	62.5	44.33±0.24**	
	31.25	37.57±0.03**	

**3f**	500	80.79±0.38^ns^	0.05
	250	77.50±0.21**	
	125	72.46±0.06**	
	62.5	63.27±0.15**	
	31.25	50.45±0.12**	

**3g**	500	83.11±0.03**	1.45
	250	65.46±0.08**	
	125	53.49±0.13**	
	62.5	41.22±0.10**	
	31.25	35.83±0.06**	

**3h**	500	77.17±0.11**	0.05
	250	74.96±0.01**	
	125	68.27±0.03**	
	62.5	58.79±0.05**	
	31.25	44.46±0.33**	

**3i**	500	73.06±0.25**	0.96
	250	52.85±0.01**	
	125	38.10±0.02**	
	62.5	30.36±0.08**	
	31.25	25.93±0.02**	

**3j**	500	78.08±0.06 ns	0.12
	250	74.97±0.01**	
	125	67.85±1.02**	
	62.5	59.01±0.00**	
	31.25	50.40±0.22**	

*Ibuprofen	500	80.06±0.02**	0.14
	250	75.94±0.02**	
	125	66.53±0.03**	
	62.5	60.55±0.20**	
	31.25	50.52±0.12**	

* Ibuprofen (500μg/ml)

** p < 0.01, p < 0.05,

ns = nonsignificant.

n = 3, in comparison to positive control, significant difference was observed in values.

**Table 6:** Antioxidant activity of synthesized of N-((2-hydroxy-3-(2-(substitutedbenzylidene)hydrazine-1-carbonyl)naphthalen-1-yl)(3-nitrophenyl/3,4-dimethoxy phenyl)methyl)acetamide derivatives (2a–j and 3a–j) (mean percentage inhibition ± SEM).

Comp.	Conc.	Mean±SEM	IC_50_

**2a**	500	86.064±0.076^**^	1.24
	250	50.333±0.020^**^	
	125	35.825±0.004^**^	
	62.5	30.254±0.009^**^	
	31.25	30.175±0.017^**^	

**2b**	500	37.499±0.007^**^	0.39
	250	36.244±0.027^**^	
	125	33.302±0.005^**^	
	62.5	32.940±0.029^**^	
	31.25	32.458±0.026^**^	

**2c**	500	83.431±0.018^**^	1.13
	250	56.519±0.037^**^	
	125	42.941±0.009^**^	
	62.5	33.371±0.031^**^	
	31.25	33.523±0.010^**^	

**2d**	500	54.756±0.008^**^	0.41
	250	47.690±0.035^**^	
	125	39.080±0.000^**^	
	62.5	35.733±0.035^**^	
	31.25	32.525±0.077^**^	

**2e**	500	62.469±0.050^**^	0.45
	250	49.107±0.000^**^	
	125	35.399±0.015^**^	
	62.5	34.555±0.022^**^	
	31.25	30.968±0.028^**^	

**2f**	500	65.346±0.018^**^	1.67
	250	48.027±0.012^**^	
	125	40.711±0.023^**^	
	62.5	31.312±0.027^**^	
	31.25	32.554±0.022^**^	

**2g**	500	87.030±0.033^**^	0.47
	250	57.466±0.067^**^	
	125	32.586±0.022^**^	
	62.5	31.348±0.067^**^	
	31.25	30.11±0.036^**^	

**2h**	500	77.912±0.036^**^	0.57
	250	52.821±0.119^**^	
	125	35.630±0.079^**^	
	62.5	31.145±0.011^**^	
	31.25	30.595±0.086^**^	

**2i**	500	53.042±0.040^**^	0.62
	250	38.089±0.050^**^	
	125	33.516±0.023^**^	
	62.5	33.905±0.080^**^	
	31.25	32.749±0.025^**^	

**2j**	500	85.643±0.104^**^	0.43
	250	62.025±0.030^**^	
	125	38.933±0.028^**^	
	62.5	30.089±0.054^**^	
	31.25	30.945±0.014^**^	

**3a**	500	92.130±0.177^**^	0.78
	250	59.262±0.123^**^	
	125	37.647±0.041^**^	
	62.5	25..95±0.027^**^	
	31.25	22.921±0.007^**^	

**3b**	500	92.592±0.025^**^	0.41
	250	72.734±0.036^**^	
	125	46.966±0.080^**^	
	62.5	34.748±0.032^**^	
	31.25	34.386±0.021^**^	

**3c**	500	70.137±0.026^**^	1.45
	250	48.995±0.065^**^	
	125	40.658±0.012^**^	
	62.5	30.002±0.012^**^	
	31.25	32.829±0.013^**^	

**3d**	500	72.329±0.012^**^	1.40
	250	39.100±0.044^**^	
	125	37.814±0.039^**^	
	62.5	32.892±0.030^**^	
	31.25	34.023±0.034^**^	

**3e**	500	61.138±0.034^**^	0.38
	250	51.174±0.011^**^	
	125	32.625±0.027^**^	
	62.5	30.585±0.057^**^	
	31.25	30.698±0.020^**^	

**3f**	500	77.094±0.01^2**^	0.49
	250	51.535±0.048^**^	
	125	34.317±0.010^**^	
	62.5	31.943±0.012^**^	
	31.25	32.601±0.013^**^	

**3g**	500	89.815±0.003^**^	0.22
	250	79.670±0.035^**^	
	125	58.864±0.032^**^	
	62.5	43.295±0.017^**^	
	31.25	31.07±0.053^**^	

**3h**	500	58.488±0.053^**^	1.58
	250	43.250±0.056^**^	
	125	38.259±0.018^**^	
	62.5	31.281±0.027^**^	
	31.25	34.425±0.020^**^	

**3i**	500	90.335±0.037^**^	0.55
	250	60.835±0.235^**^	
	125	39.404±0.245^**^	
	62.5	31.945±0.052^**^	
	31.25	30.876±0.042^**^	

**3j**	500	30.999±0.011^**^	0.20
	250	68.988±0.276^**^	
	125	42.281±0.076^**^	
	62.5	30.363±0.021^**^	
	31.25	23.279±0.026^**^	

^*^ **Ascorbic acid**	500	99.921±0.029	0.48
	250	88.951±0.001	
	125	74.336±0.236	
	62.5	55.320±0.299	
	31.25	39.788±0.413	

**Table 7:** Docking affinities of N-((2-hydroxy-3-(2-(substitutedbenzylidene)hydrazine-1-carbonyl)naphthalen-1-yl)(3-nitrophenyl/3,4-dimethoxyphenyl)methyl)acetamide derivatives (2a–j and 3a–j) with their respective targets.

Comp.	Binding energy (kcal/mol)			
	PDB:5H67	PDB:5TZ1	PDB:4Z69	PDB:1DXO
**2a**	−8.4	−9.3	−10.9	−8.8
**2b**	−8.8	−10.3	−9.2	−8.7
**2c**	−8.4	−9.0	−9.8	−8.9
**2d**	−8.9	−9.1	−10.6	−8.9
**2e**	−9.3	−9.8	−10.5	−9.3
**2f**	−9.4	−9.1	−10.1	−9.6
**2g**	−9.2	−9.0	−9.9	−9.5
**2h**	−8.6	−8.9	−9.6	−8.9
**2i**	−8.8	−9.4	−9.6	−9.0
**2j**	−8.4	−9.1	−9.8	−8.8
**3a**	−8.4	−8.5	−9.6	−7.8
**3b**	−7.7	−8.5	−9.7	−8.4
**3c**	−7.9	−8.0	−9.3	−7.5
**3d**	−8.0	−8.3	−7.9	−8.2
**3e**	−7.9	−7.7	−9.8	−8.0
**3f**	−7.9	−9.5	−10.0	−8.0
**3g**	−8.7	−8.8	−9.8	−7.9
**3h**	−7.9	−8.4	−9.6	−8.2
**3i**	−8.4	−8.6	−9.3	−8.2
**3j**	−8.2	−8.1	−9.0	−7.8

**Table 8:** Molecular docking interactions of compound **3i** and **3d** with target residues of PDB:5H67.

Comp.	Functional groups	Interactions	Amino acids	Bond length (Å)
**3i** (Most potent)	Oxygen of C=O group bound to hydrazine and oxygen of acetamide	Three hydrogen bonds	ArgA:45 and SerA:142	2.96, 3.05 and 2.74
	Hydrogen of hydrazide	Hydrogen bond	SerA:55	2.48
	Ring: D	Pi-cation and pi-anion	Glu:163 and Lys:145	3.73 and 3.87
	Ring: B	Pi-sigma and pi-alkyl	ArgA:57	3.42, 4.75
**3d** (Second most potent)	Hydrogen of hydrazide	Hydrogen bond	GlyA:132	2.66
	Ring: A	Three pi-alkyl	ArgB:1109, AlaA:137	3.40 and 4.40
	3-Methoxy of ring: B	Carbon-hydrogen bond	PheA:90	3.73
	Ring: D	Pi-anion	GluA:136	3.78

**Table 9:** Molecular docking interactions of compounds 3i and 3h with target residues of PDB 5TZ1,

Comp.	Functional groups	Interactions	Amino acids	Bond length (Å)
**3i** (Most potent)	Hydrogen of OH linked to ring: A Oxygen of carbonyl group of acetamide	2 Hydrogen bond	GluA:194 SerA:222	2.39 2.10
	Hof Hydrazide	Salt bridge, Charge-charge	GluA:194	1.98
	Ring: C	Pi-anion	AspA:225	4.49
	Ring: A	Pi-pi stacked	TyrA:221	5.88
	Ring: A	2 Pi-alkyl	IleA:197 AlaA:218	4.76 4.96
	Ring: C	1 Pi-alkyl	ProA:193	5.48
**3h** (Second most potent)	Oxygen of OCH_3_ of ring: B Hydrogen of OH linked to ring: A Oxygen of carbonyl group of acetamide	3 Hydrogen bond	ThrA:172 GluA:194 SerA:322	2.67 2.19 2.00
	Carbon of OCH_3_ linked to ring: C	Carbon-hydrogen bond	MetA:189	3.70
	Ring: C	Pi-sulfur	Met:79	4.11
	Ring: C	3 Pi-alkyl	LysA:190	3.70
	Ring: A		AlaA:218 IleA:197	4.62 4.93
	Hydrogen of hydrazide	Salt bridge, Charge-Charge	GluA:194	2.04

**Table 10:** Molecular docking interactions of compounds 3h and 3f with target residues of PDB 4Z69.

Comp.	Functional groups	Interactions	Amino acids	Bond length (Å)
**3h** (Most potent)	Oxygen of acetamide	Hydrogen bond	LysA:199	1.99
	Nitrogen of hydrazide group	Charge-Charge	GluA:153	3.47
	Ring: A	2 Pi-sulfur	CysA:245, CysA:253	4.87 and 5.18
	Ring: C	Pi-cation	ArgA:257	3.80
	Ring: A	Pi-pi T shaped	TyrA:150	5.22
	Carbon of acetamide	Pi-sigma	HisA:242	3.87
	Ring: C	Pi-alkyl	AlaA:291	4.38
	Oxygen of OCH_3_ linked to ring: C	Carbon hydrogen bond	SerA:287	3.36
**3f** (Second most potent)	Oxygen linked to hydrazide Oxygen of OCH_3_ linked to ring: C	Two hydrogen bonds	TyrA:161 and ArgA: 145	2.55 and 2.65
	Ring: A	Pi-cation	HisA: 146	4.09
	Ring: B	2 Pi-pi stacked	TyrA:161 and TyrA:138	4.97 3.89
	Chloro of ring: B	Three alkyl	ArgA:117, ProA:118 and IleA:142	4.25, 5.16 and 5.09
	Chloro of ring: B	Pi-sigma	TyrA:161	3.52
	Ring: A	Three pi-alkyl	LysA:190, ArgA:186	4.20, 5.33 and 4.71
	Ring: C	Pi-alkyl	ArgA:114	5.00
	Chloro of ring: B	Pi-alkyl	TyrA: 138	4.21
	Ring: C	2 Carbon-hydrogen bonds	ArgA:114, LeuA:115	3.41, 3.00

**Table 11:** Molecular docking interactions of compounds 3j and 3g with target residues of PDB 1DXO.

Comp.	Functional groups	Interactions	Amino acids	Bond length (Å)
**3j** (Most potent)	Oxygen of carbonyl group bound to hydrazine and hydrogen of hydroxyl group linked to ring: A	Two hydrogen bonds	GlyA:174, TyrA:132	2.77 and 1.97
	Carbon of ring: D	Two carbon hydrogen bonds	GluA:117	3.65 and 3.53
	2-Hydoxy substituted ring: A	Two pi-pi stacking	PheA:178	5.15 and 4.01
	Ring: B	Pi-pi-T shaped	TrpA:169	4.98
**3g** (Second most potent)	Oxygen of C=O group bound to hydrazine and oxygen of Ring: D	Two hydrogen bonds	MetA:238 and TrpA:169	2.85 and 2.70
	Carbon of acetamide and ring: B	Pi-sigma	PheA:228 and LeuA:259	3.68 and 3.47
	Ring: A	Pi-pi stacking	Phe:236	5.03

**Table 12:** Lipinski’s rule of five calculations: topological polar surface area (TPSA), aqueous solubility, and number of rotatable bonds for the synthesized N-((2-hydroxy-3-(2-(substitutedbenzylidene)hydrazine-1-carbonyl)naphthalen-1-yl)(3-nitrophenyl/3,4-dimethoxy phenyl)methyl)acetamide derivatives (2a–j and 3a–j).

Comp.	miLog P	Log S (mol/L)	TPSA (Å^2^)	MW	nON	nOHNH	nvoilation	Nrot	Vol
**2a**	5.04	−7.369	182.44	527.49	12	3	3	8	444.96
**2b**	4.62	−6.613	156.84	498.50	10	4	0	7	429.64
**2c**	5.14	−6.945	155.08	542.55	11	3	3	9	472.72
**2d**	4.75	−6.945	155.08	542.55	11	3	3	9	472.72
**2e**	6.36	−8.381	136.61	551.39	9	3	2	7	448.70
**2f**	6.36	−8.381	136.61	551.39	9	3	2	7	448.70
**2g**	6.38	−8.381	136.61	551.39	9	3	2	7	448.70
**2h**	4.44	−6.631	166.07	528.52	11	4	2	8	445.19
**2i**	4.82	−6.931	166.07	542.55	11	4	2	9	471.99
**2j**	4.73	−6.963	164.31	572.57	12	3	2	10	498.26
**3a**	4.75	−6.945	155.08	542.55	11	3	2	9	470.72
**3b**	4.33	−6.189	129.48	513.55	9	4	1	8	457.40
**3c**	4.85	−6.521	127.72	557.60	10	3	1	10	500.47
**3d**	4.46	−6.521	127.72	557.60	10	3	1	10	500.47
**3e**	6.07	−7.957	109.26	566.44	8	3	2	8	476.45
**3f**	6.07	−7.957	109.26	566.44	8	3	2	8	476.45
**3g**	6.10	−7.957	109.26	566.44	8	3	2	8	476.45
**3h**	4.15	−6.207	138.72	543.58	10	4	1	9	482.95
**3i**	4.53	−6.507	138.72	557.60	10	4	1	10	499.75
**3j**	4.44	−6.539	136.96	587.63	11	3	2	11	526.02

miLogP: Molinspiration calculated partition coefficient,

Log S (mol/L): Logarithm of the aqueous solubility,

TPSA (Å^2^): Topological Polar Surface Area,

MW: Molecular Weight,

nON: Number of oxygen and nitrogen atoms,

nOHNH: Number of hydroxyl (OH) and amine (NH) groups,

nviolations: Number of violations of Lipinski’s Rule of Five,

Nrot: Number of rotatable bonds,

Vol: Molecular volume.

**Table 13:** Standard values of ADME parameters.

Remarks	Human intestinal absorption	Caco-2 cell permeability	MDCK cell permeability	Plasma protein binding	Blood-brain barrier penetration
**Well/high**	70–100%	>70	>500	>90	>2.0
**Moderate**	20–7%	4–70	25–500	-	0.1–2.0
**Poor/low**	0–20%	<4	<25	<90%	<0.1

**Table 14:** ADME property values of synthesized N-((2-hydroxy-3-(2-(substitutedbenzylidene)hydrazine-1-carbonyl)naphthalen-1-yl)(3-nitrophenyl/3,4-dimethoxy phenyl)methyl)acetamide derivatives (2a–j and 3a–j) using the Pre-ADMET online server.

Comp.	Human intestinal absorption (HIA, %)	In vitro Caco-2 cell permeability (nm/s)	In vitro MDCK cell permeability (nm/s)	In vitro plasma protein binding (%)	In vivo blood brain-barrier penetration (C.brain/C. blood)	Pgp_inhibition
**2a**	88.11	20.30	0.07	95.46	0.03	Inhibitor
**2b**	90.18	20.00	0.09	93.71	0.05	Inhibitor
**2c**	94.02	19.23	0.05	91.47	0.02	Inhibitor
**2d**	94.02	19.56	0.06	92.65	0.03	Inhibitor
**2e**	94.79	16.49	0.04	98.96	0.13	Inhibitor
**2f**	94.78	15.07	0.04	98.68	0.12	Inhibitor
**2g**	94.79	17.09	0.05	100	0.14	Inhibitor
**2h**	90.20	19.53	0.07	91.01	0.04	Inhibitor
**2i**	90.51	18.45	0.06	89.54	0.06	Inhibitor
**2j**	94.07	19.26	0.05	91.95	0.04	Inhibitor
**3a**	94.02	17.32	0.05	92.97	0.02	Inhibitor
**3b**	92.67	20.63	0.05	89.19	0.52	Inhibitor
**3c**	94.32	23.21	0.04	90.22	0.05	Inhibitor
**3d**	94.32	23.43	0.05	90.88	0.06	Inhibitor
**3e**	96.15	22.36	0.04	87.29	0.84	Inhibitor
**3f**	96.15	21.98	0.04	87.37	0.73	Inhibitor
**3g**	96.15	22.50	0.05	87.14	0.94	Inhibitor
**3h**	92.03	20.46	0.05	89.34	0.13	Inhibitor
**3i**	91.72	20.23	0.02	86.04	0.13	Inhibitor
**3j**	94.10	25.05	0.04	89.88	0.04	Inhibitor

Caco-2: Cells derived from human colon adenocarcinomas; MDCK: Medin–Darbey canine kidney epithelial cells; Pgp: P-glycoprotein (plasma membrane protein).

**Table 15:** Bioactivity and toxicity risk of synthesized N-((2-hydroxy-3-(2-(substitutedbenzylidene)hydrazine-1-carbonyl)naphthalen1-yl)(3-nitrophenyl/3,4-dimethoxy phenyl)methyl)acetamide derivatives (2a–j and 3a–j).

Comp.	GPCR ligand	Ion-channel modu-Lator	Kinase inhibi-tor	Nuclear recep-tor ligand	Prot-ease inhibi-tor	Mutagenic	Reproductive effective	Irritant
**2a**	−0.31	−0.73	−0.48	−0.54	−0.38	Low	None	None
**2b**	−0.31	−0.66	−0.47	−0.50	−0.40	Low	None	None
**2c**	−0.33	−0.88	−0.55	−0.57	−0.40	Low	None	None
**2d**	−0.34	−0.84	−0.53	−0.60	−0.43	Low	None	None
**2e**	−0.34	−0.71	−0.52	−0.58	−0.44	Low	None	None
**2f**	−0.31	−0.71	−0.51	−0.56	−0.42	Low	Low	None
**2g**	−0.31	−0.69	−0.50	−0.55	−0.42	Low	None	None
**2h**	−0.34	−0.78	−0.50	−0.57	−0.45	Low	None	None
**2i**	−0.36	−0.84	−0.55	−0.58	−0.45	Low	None	None
**2j**	−0.38	−0.97	−0.60	−0.72	−0.42	Low	None	None
**3a**	−0.34	−0.84	−0.53	−0.60	−0.43	Low	None	None
**3b**	−0.23	−0.69	−0.39	−0.47	−0.34	Low	None	None
**3c**	−0.24	−0.91	−0.50	−0.56	−0.30	Low	None	None
**3d**	−0.25	−0.85	−0.47	−0.56	−0.32	Low	None	None
**3e**	−0.26	−0.76	−0.46	−0.55	−0.38	Low	None	None
**3f**	−0.23	−0.75	−0.45	−0.53	−0.36	Low	Low	None
**3g**	−0.23	−0.74	−0.44	−0.52	−0.33	Low	None	None
**3h**	−0.23	−0.79	−0.43	−0.52	−0.33	Low	None	None
**3i**	−0.28	−0.88	−0.50	−0.58	−0.37	Low	None	None
**3j**	−0.31	−1.02	−0.58	−0.71	−0.33	Low	None	None

## Footnotes

1 Molinspiration Cheminformatics, Calculation of Molecular Properties and Bioactivity Score. [online].Website: https://www.molinspiration.com [accessed 12 May 2022].

2 BMDRC. PreADMET: Prediction of ADME/Tox. [online]. Website: https://preadmet.webservice.bmdrc.org [accessed 12 May 2022].
