# Supplementary material for: Synthesis, characterization, antimicrobial, antioxidant, and antiinflammatory evaluation and molecular docking studies of N-((2-hydroxy-3-(2-(substitutedbenzylidene)hydrazine-1-carbonyl)naphthalen-1-yl)(3-nitrophenyl/3,4-dimethoxyphenyl)methyl)acetamide derivatives
Source: Turk J Chem. 2025 Apr 18;49(6):683–705. doi: 10.55730/1300-0527.3764 (PMC12779050; doi:10.55730/1300-0527.3764)

Supporting Information

Compound 2a : IR Spectra

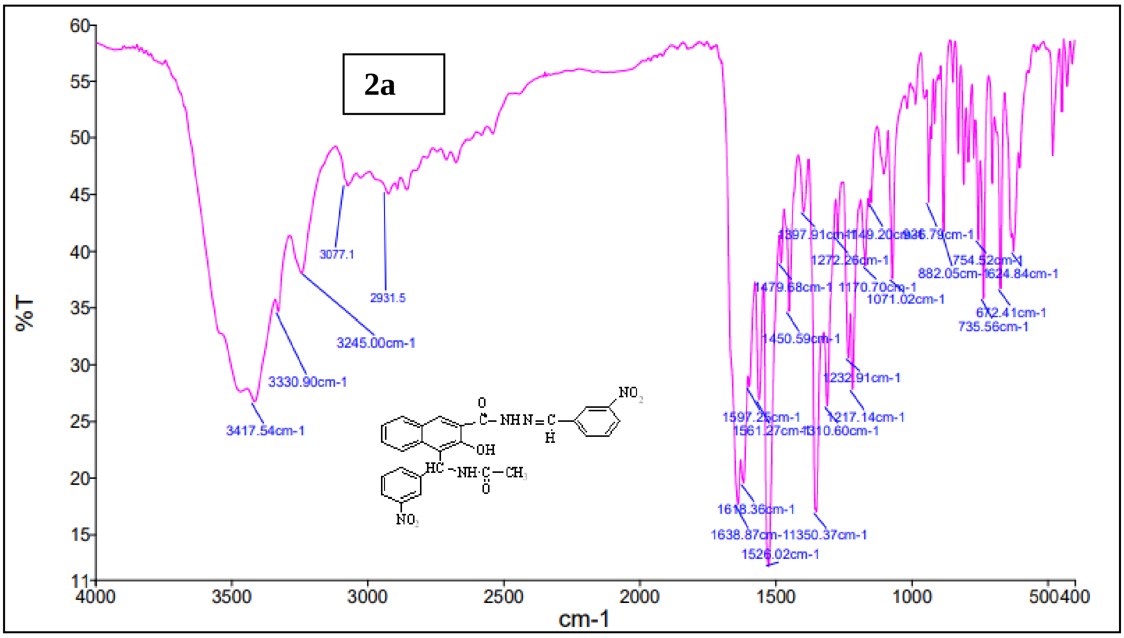

Compound 2b : IR Spectra

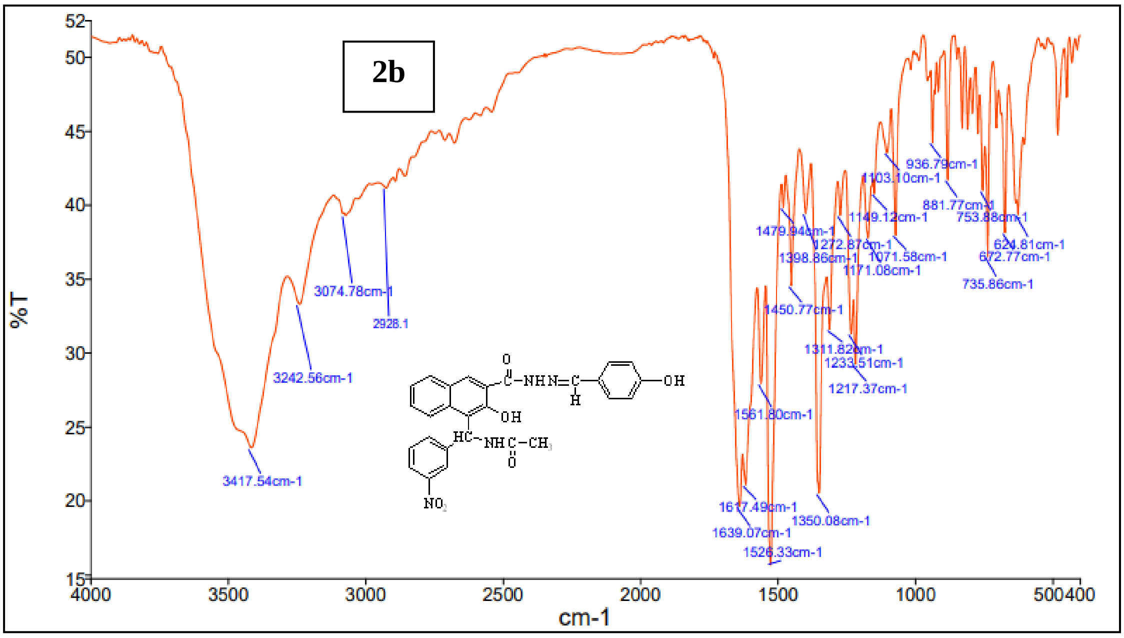

<u>Compound 2c</u> : IR Spectra

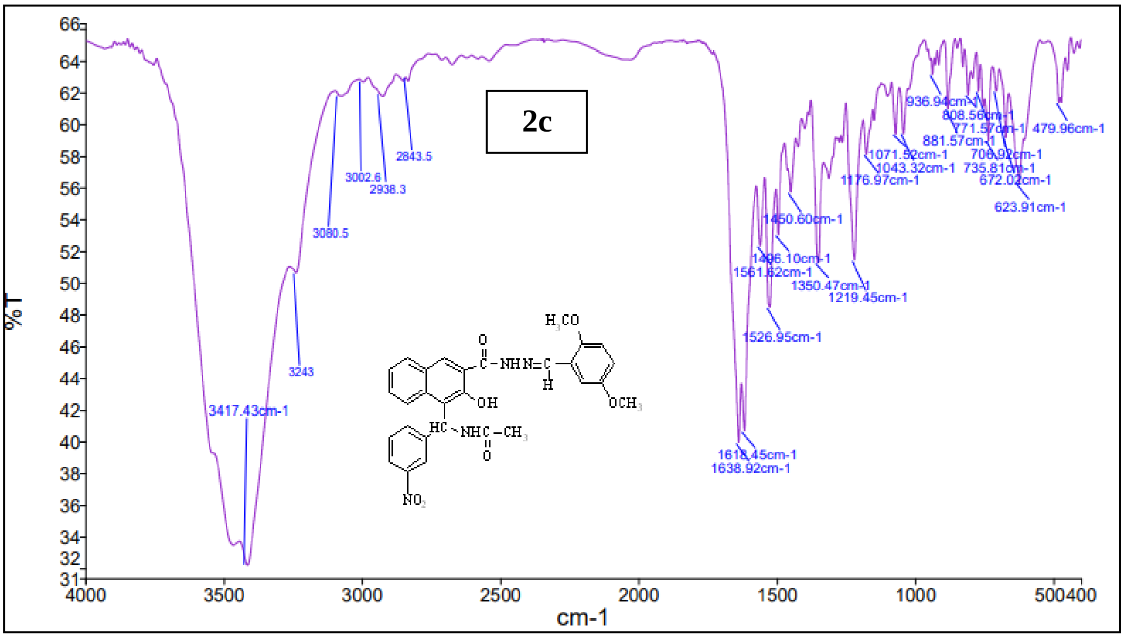

Mass Spectra

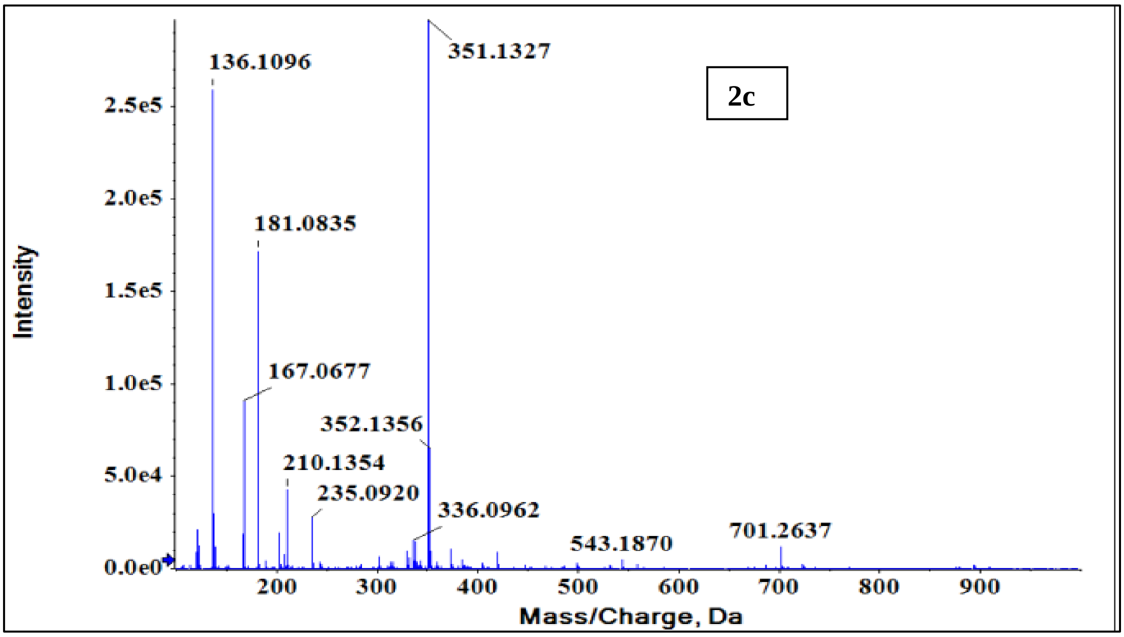

^{1}H NMR Spectra

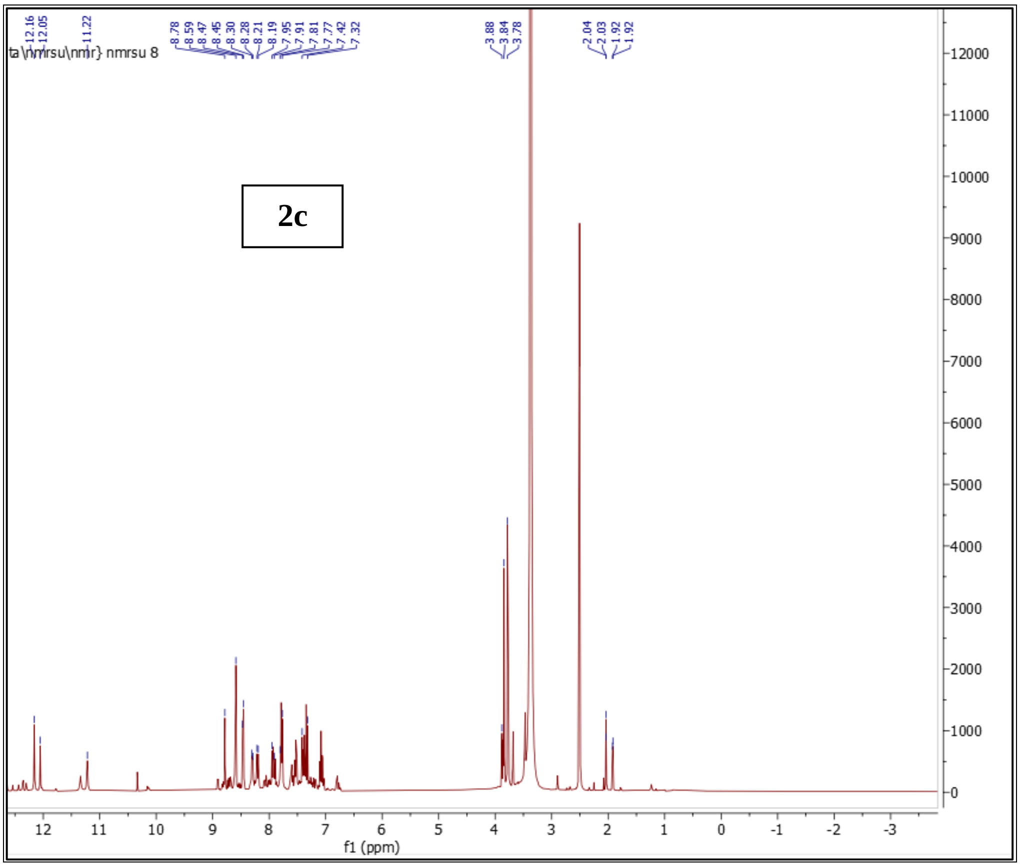

^{13}C NMR Spectra

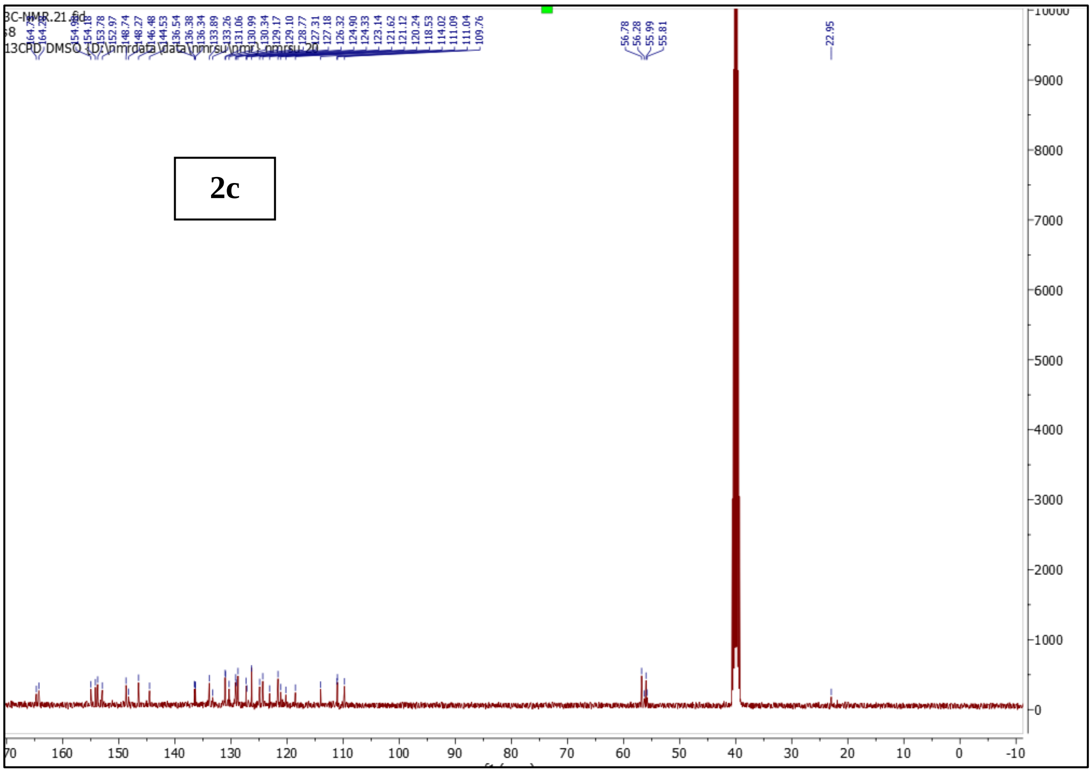

Compound 2d: IR Spectra

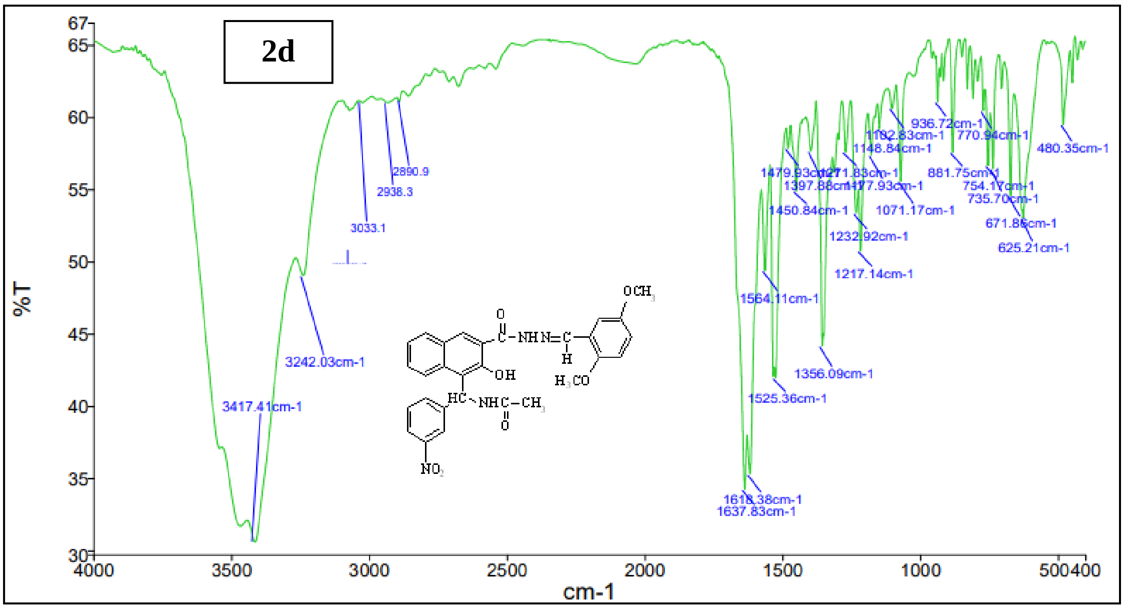

Compound 2e: IR Spectra

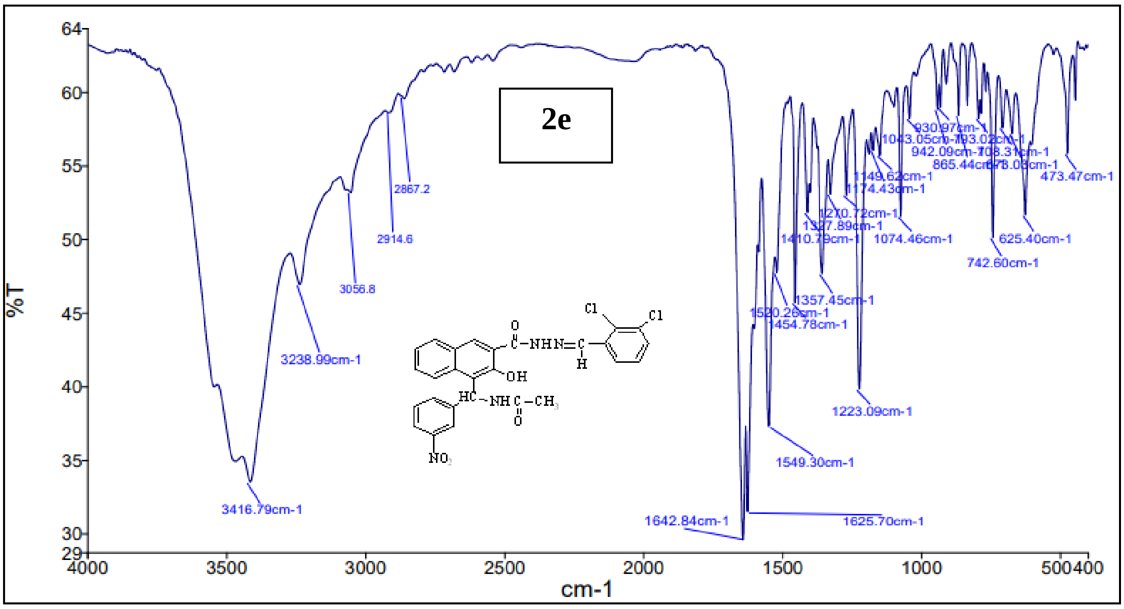

<u>Compound 2f</u> : ^{1}H NMR Spectra

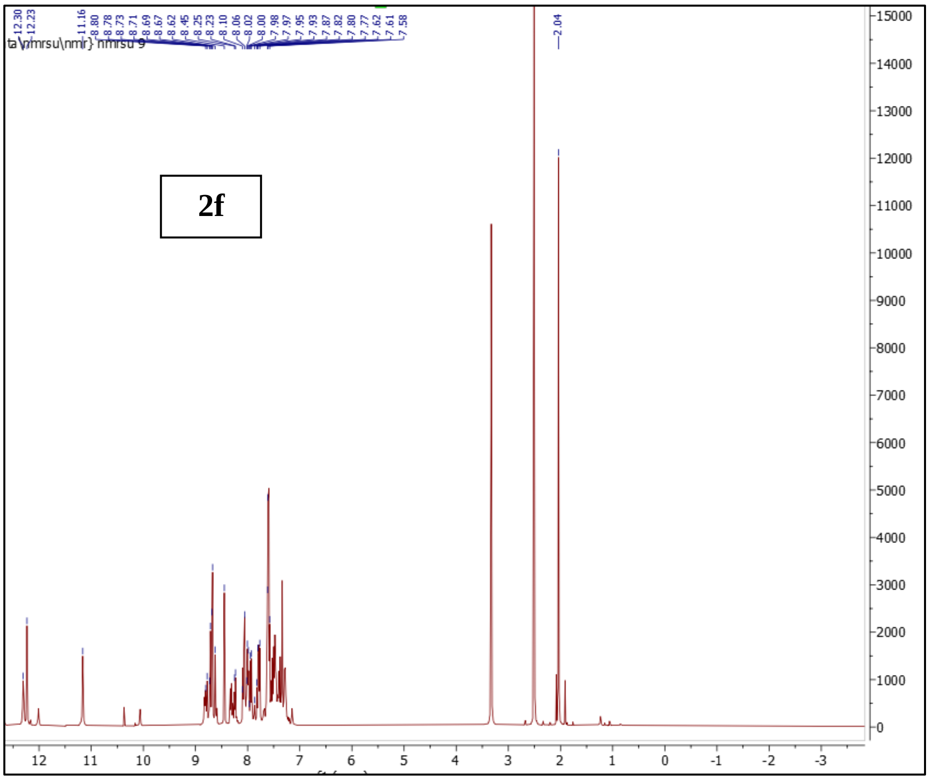

^{13}C NMR Spectra

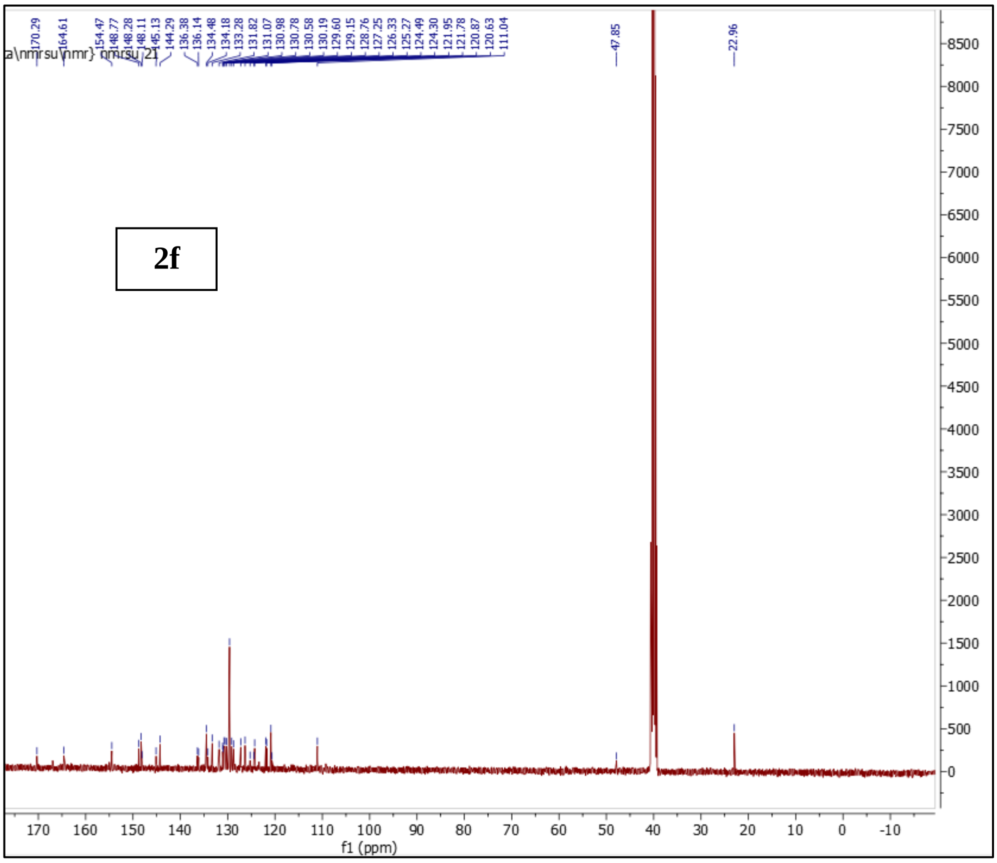

Compound 2j : ^{1}H NMR Spectra

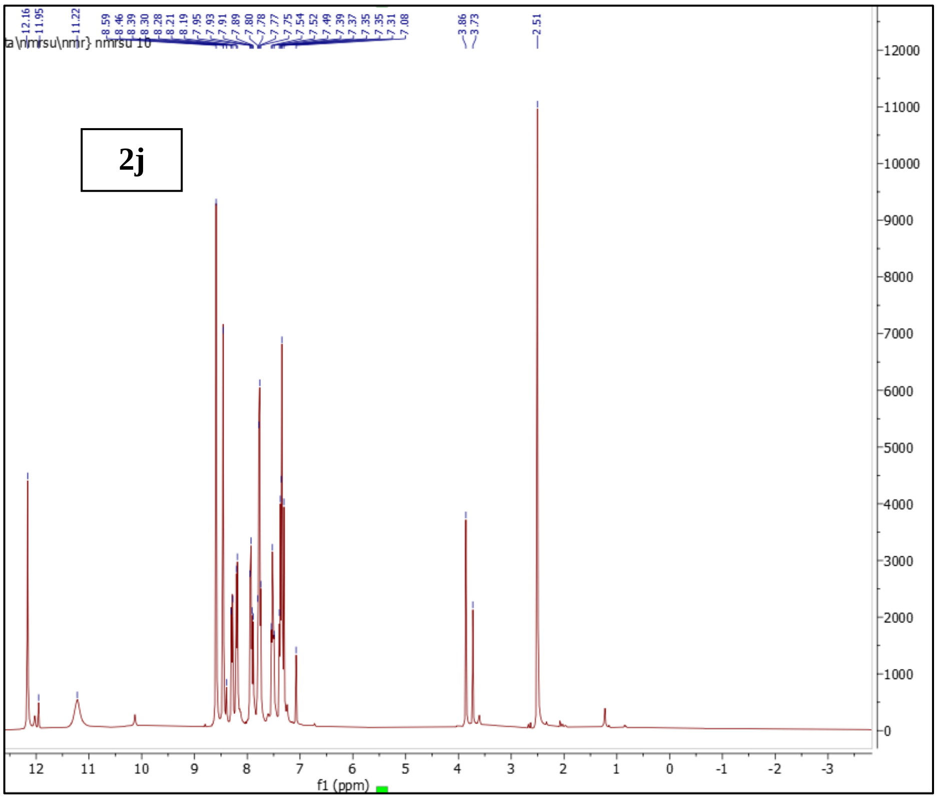

^{13}C NMR Spectra

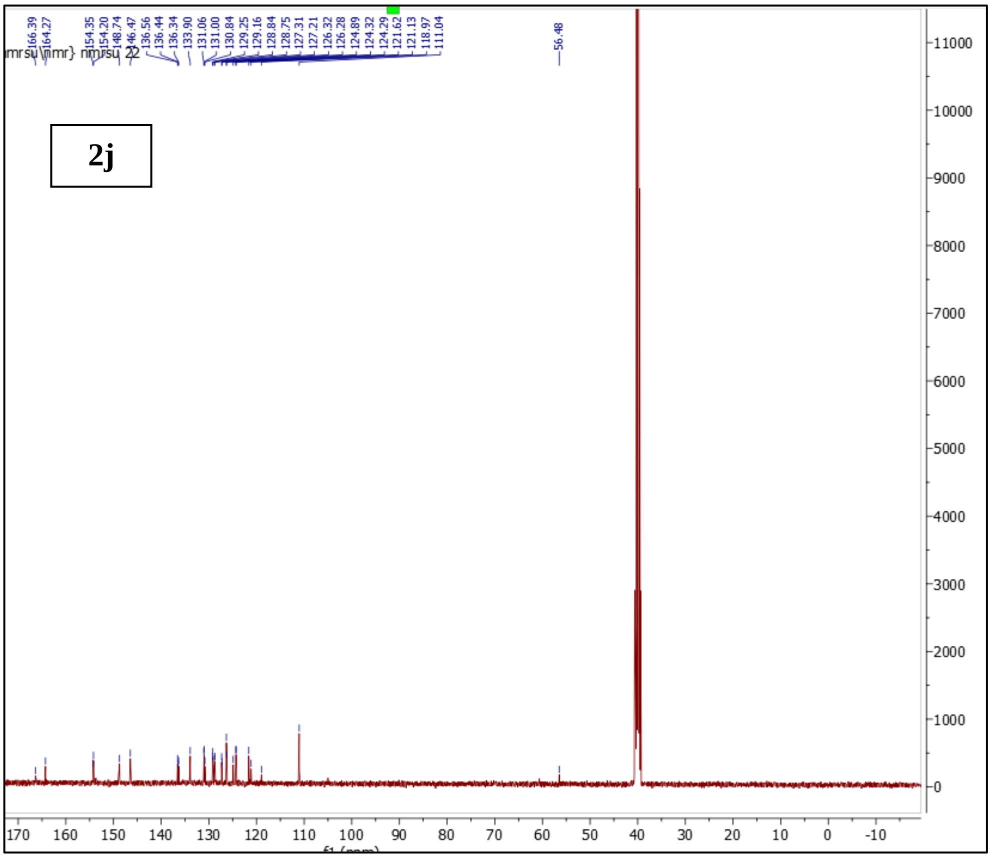

Compound 3a : IR Spectra

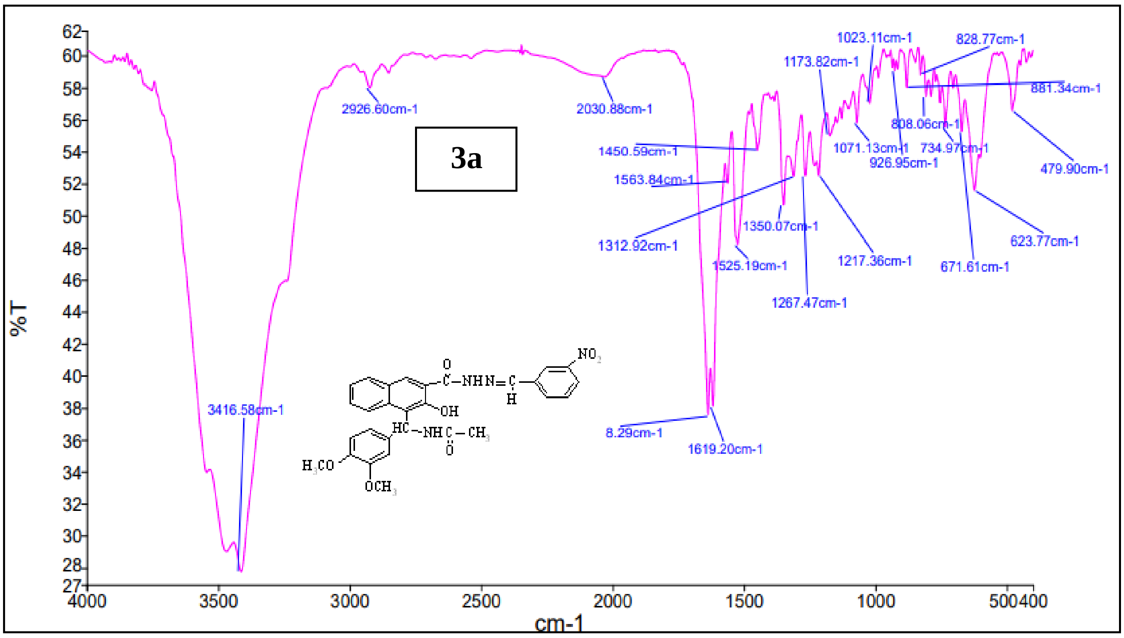

Compound 3b : IR Spectra

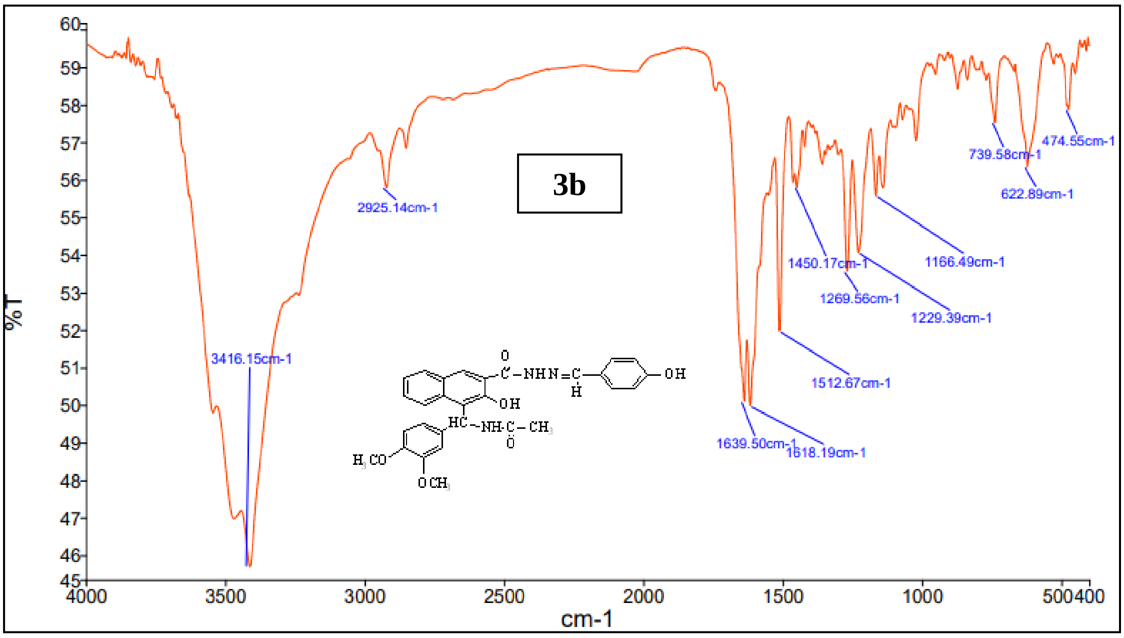

Compound 3c : IR Spectra

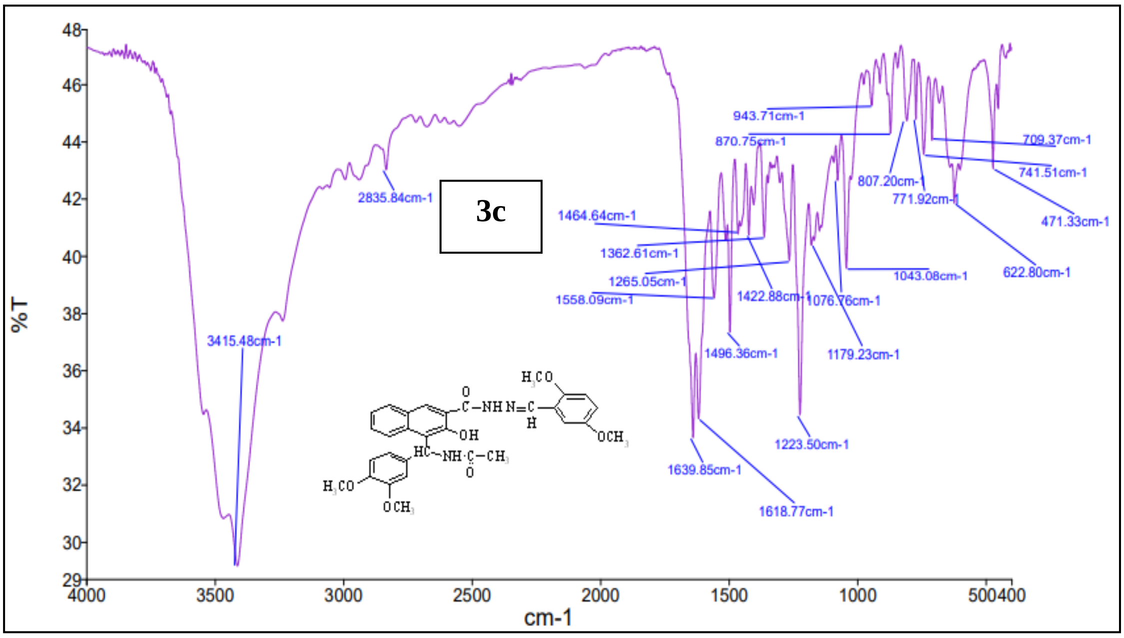

Compound 3d : IR Spectra

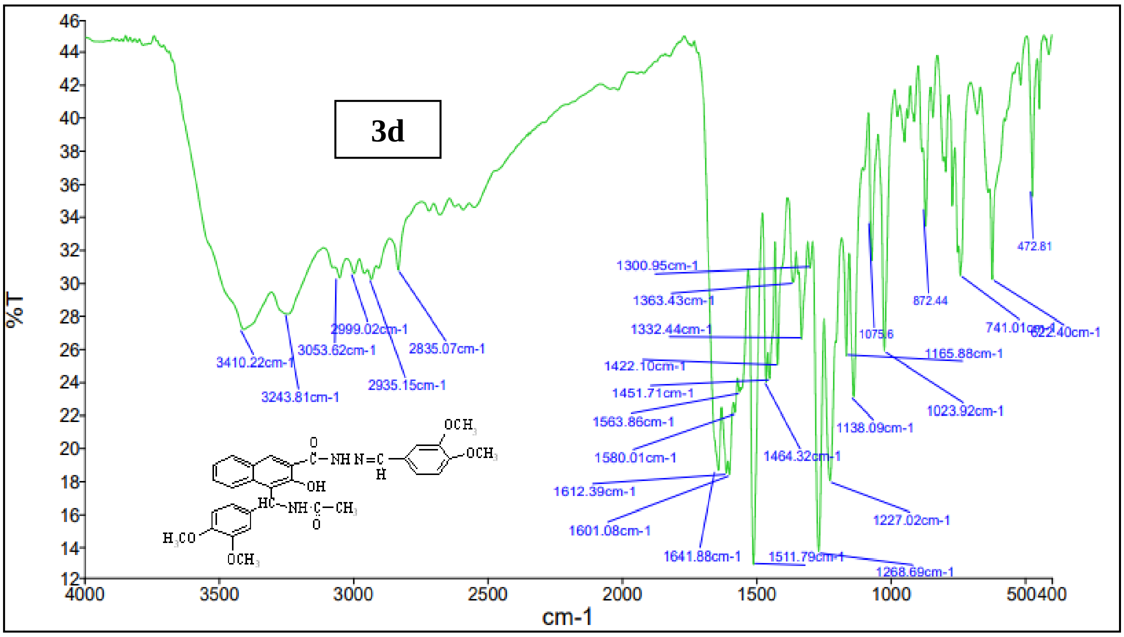

Compound 3e : IR Spectra

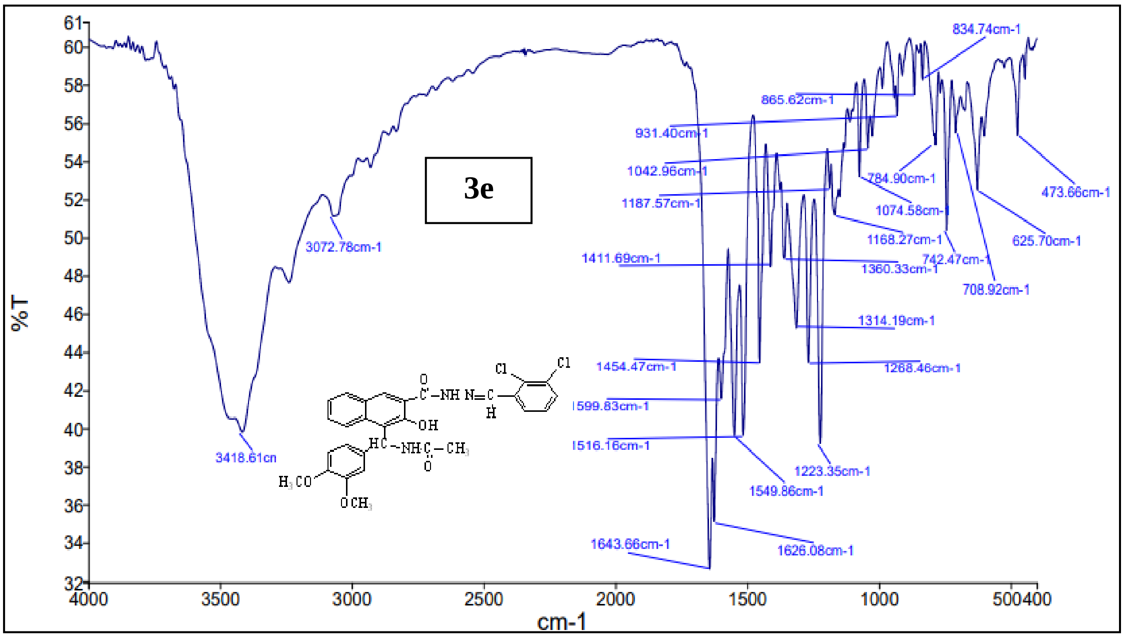

Compound 3f : IR Spectra

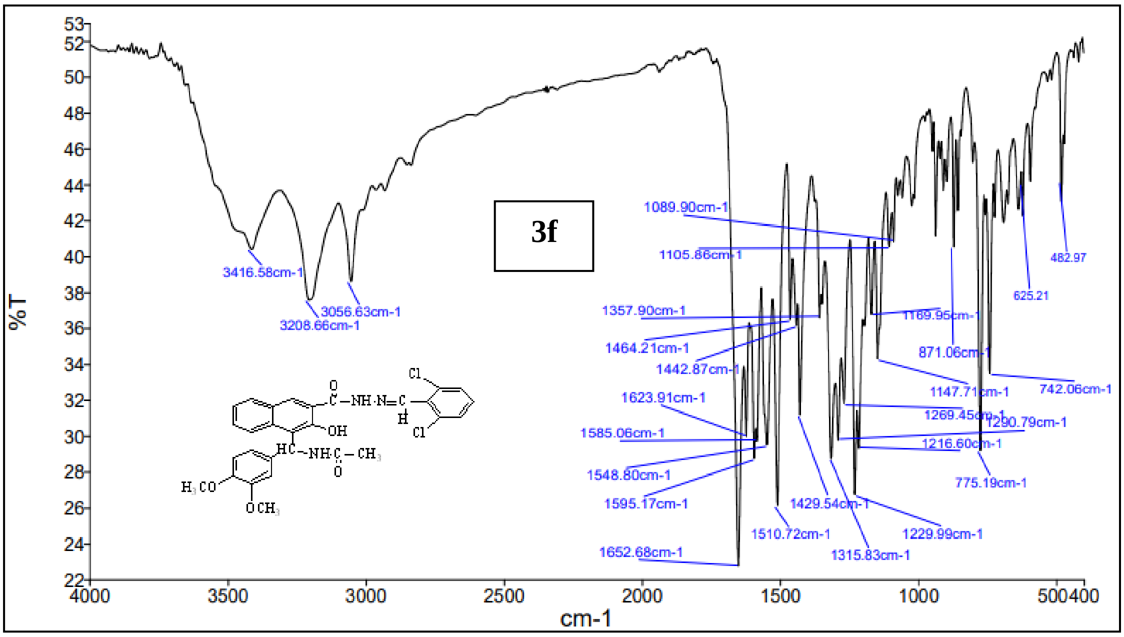

Compound 3g : IR Spectra

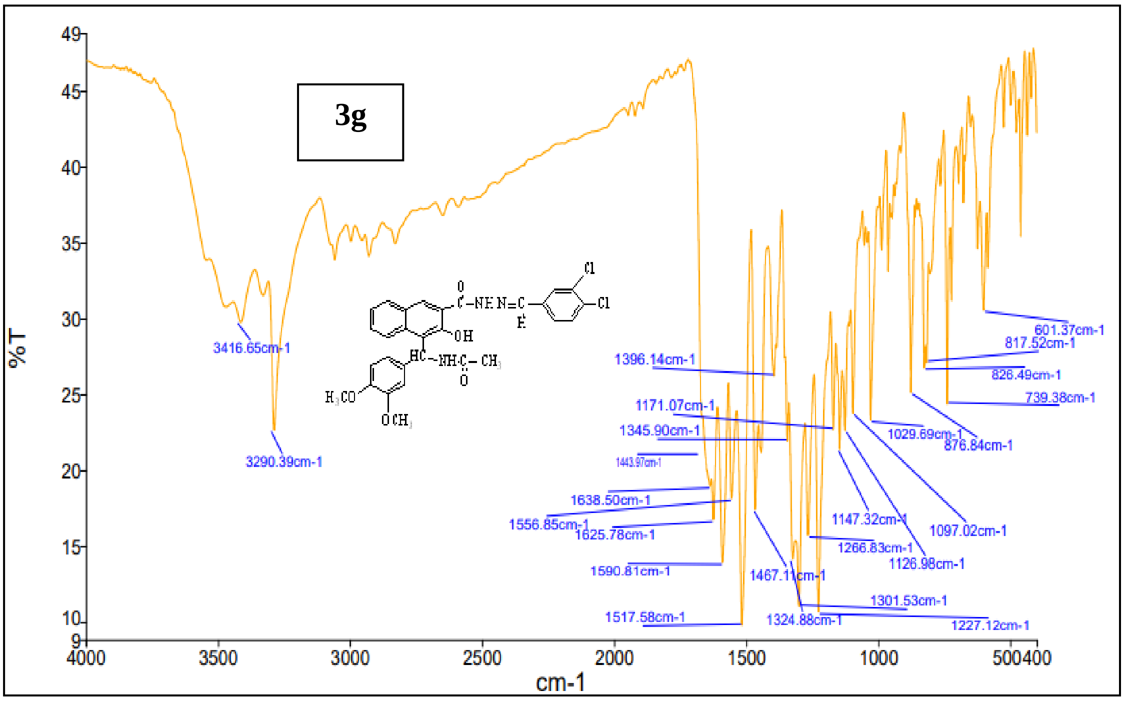

^{1}H NMR Spectra

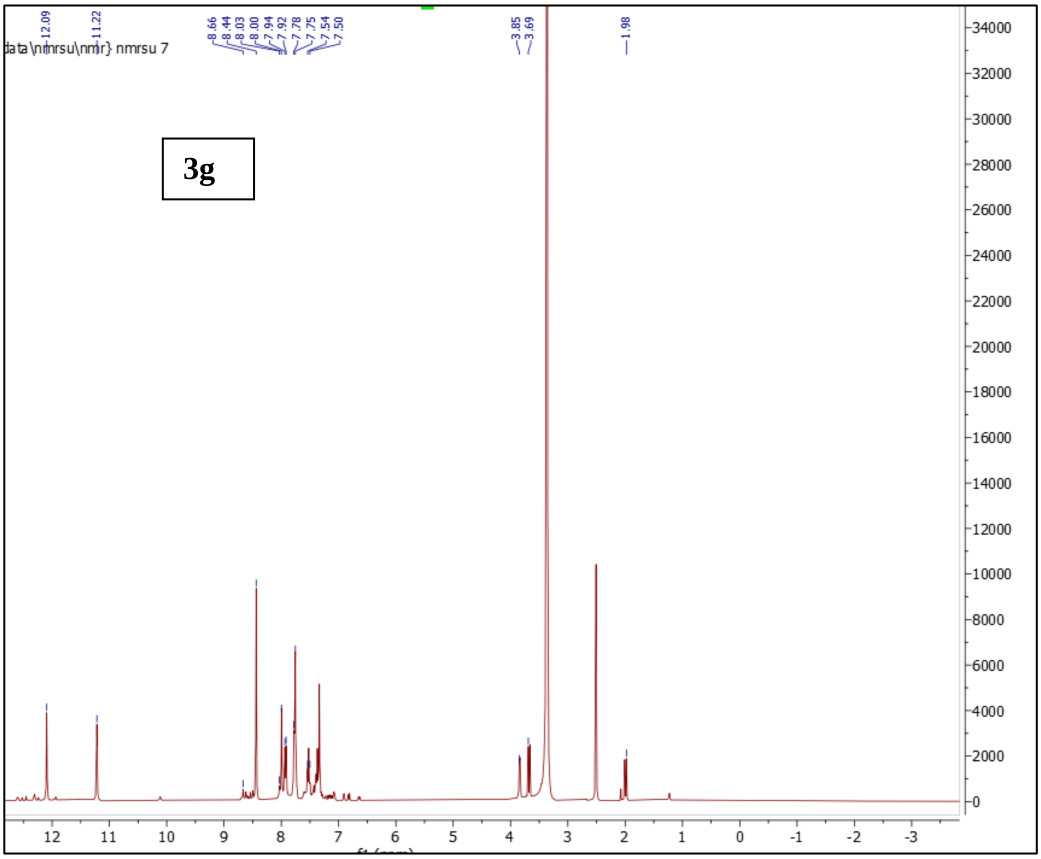

^{13}C NMR Spectra

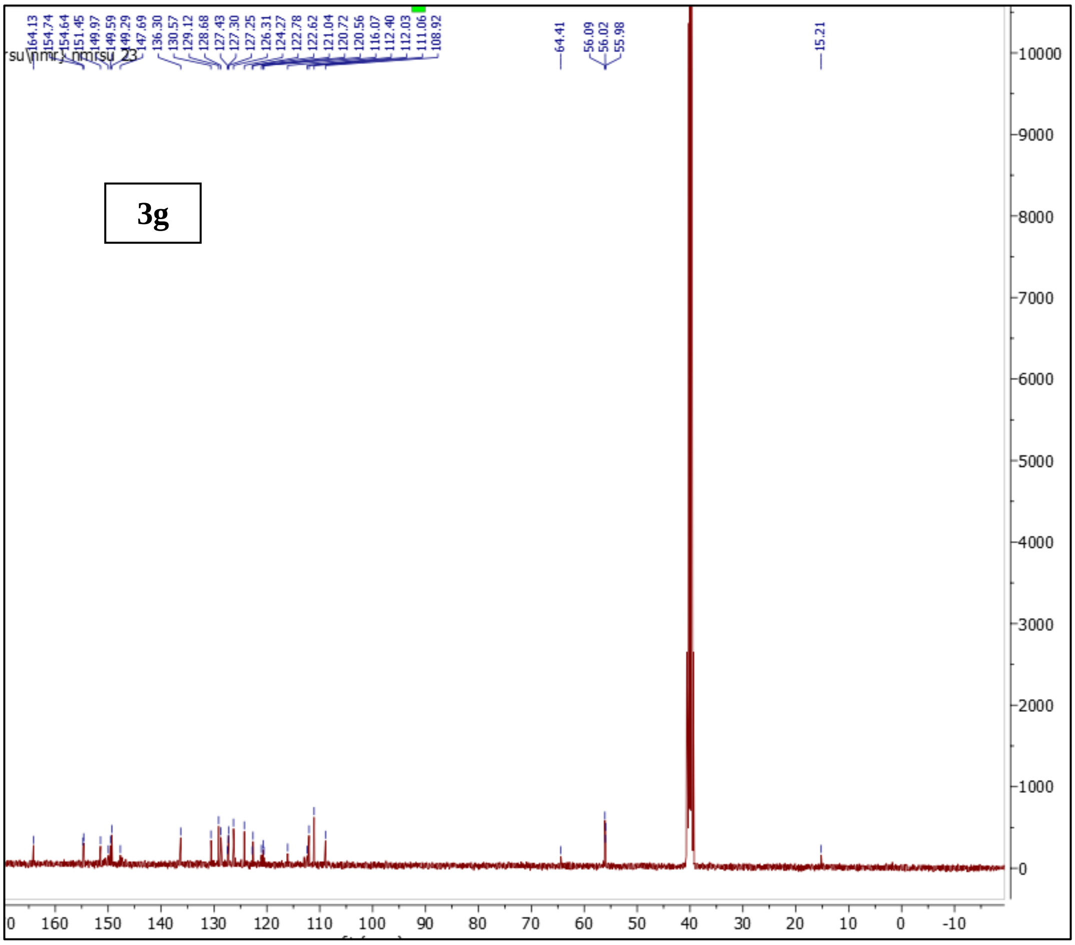

Compound 3h: IR Spectra

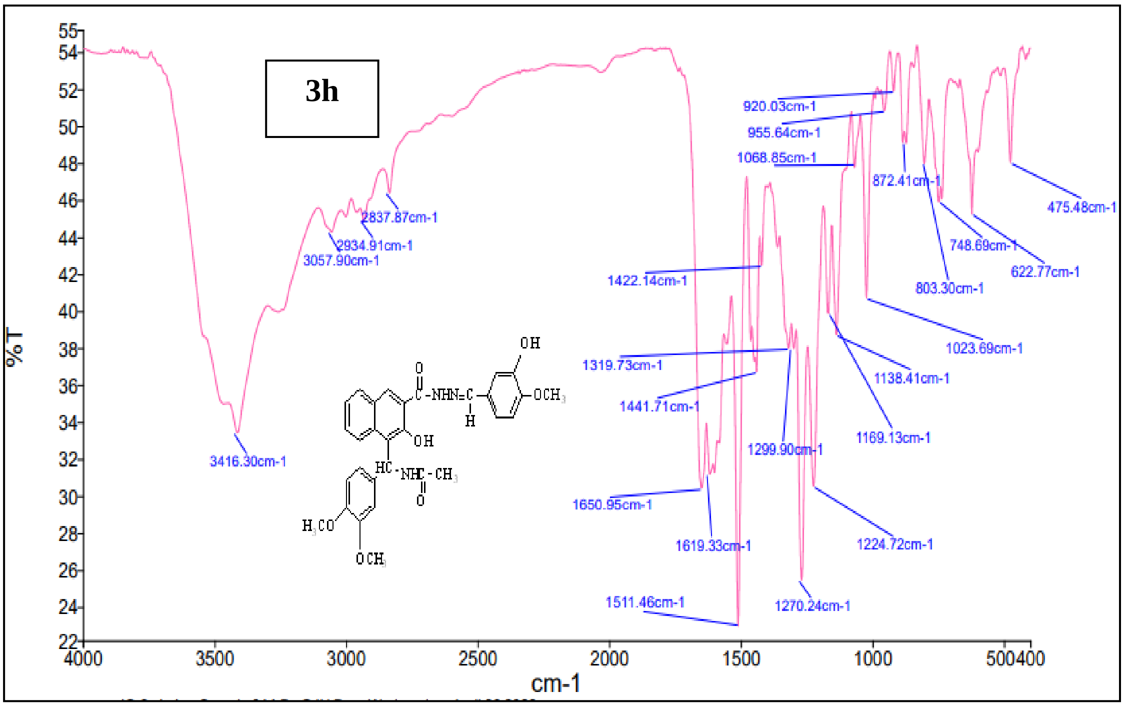

<u>Compound 3i</u> : IR Spectra

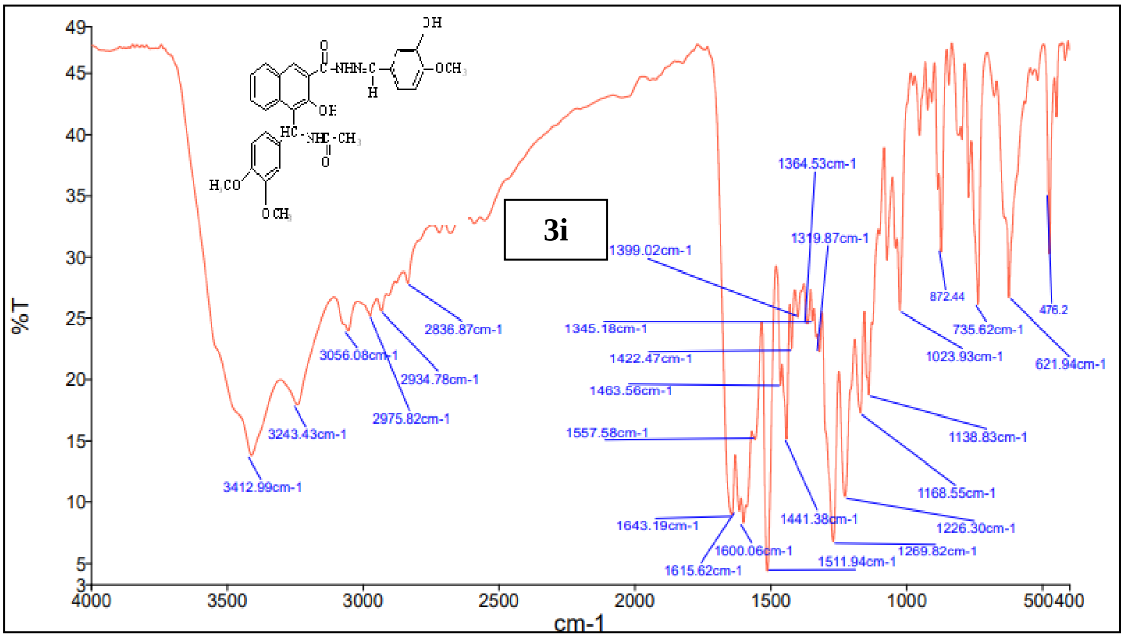

Mass Spectra

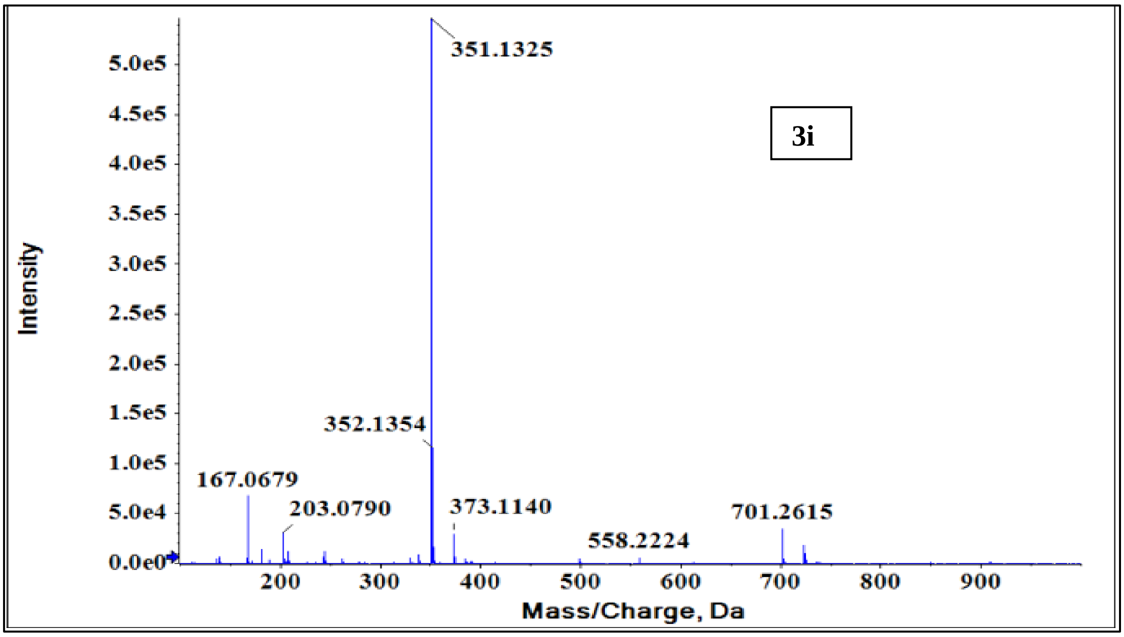

^{1}H NMR Spectra

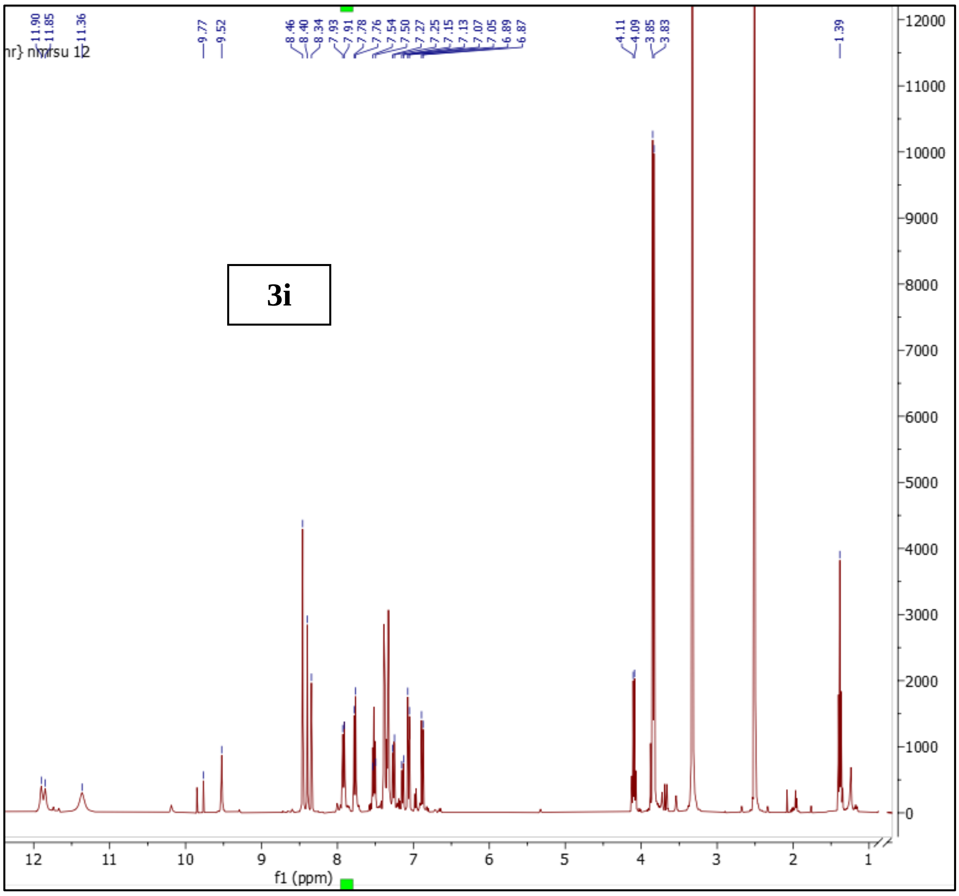

^{13}C NMR Spectra

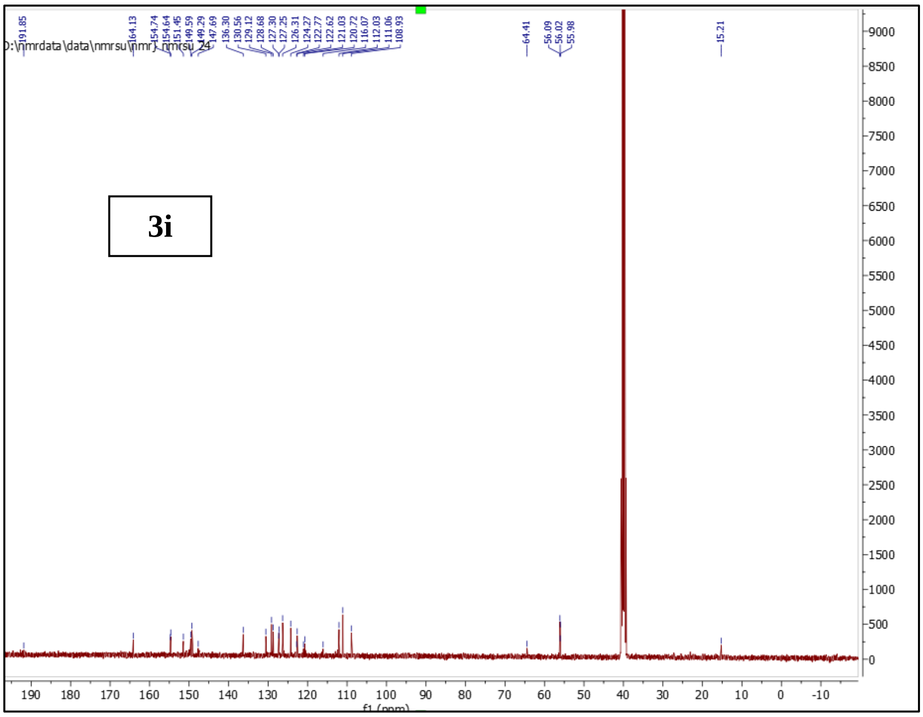

Compound 3j : IR Spectra

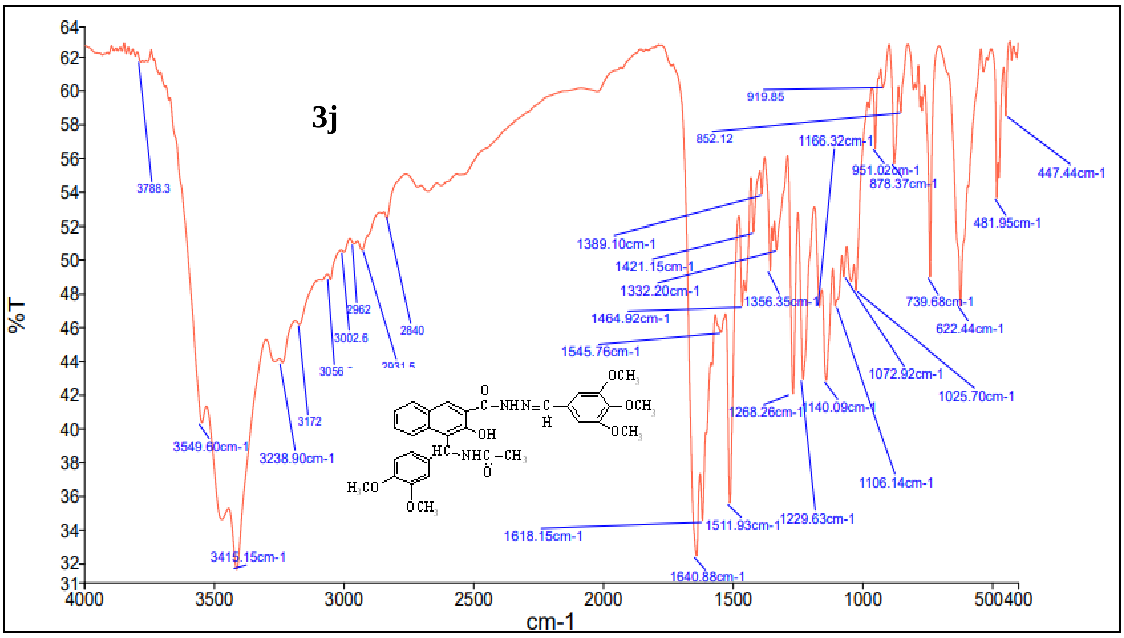

Mass Spectra

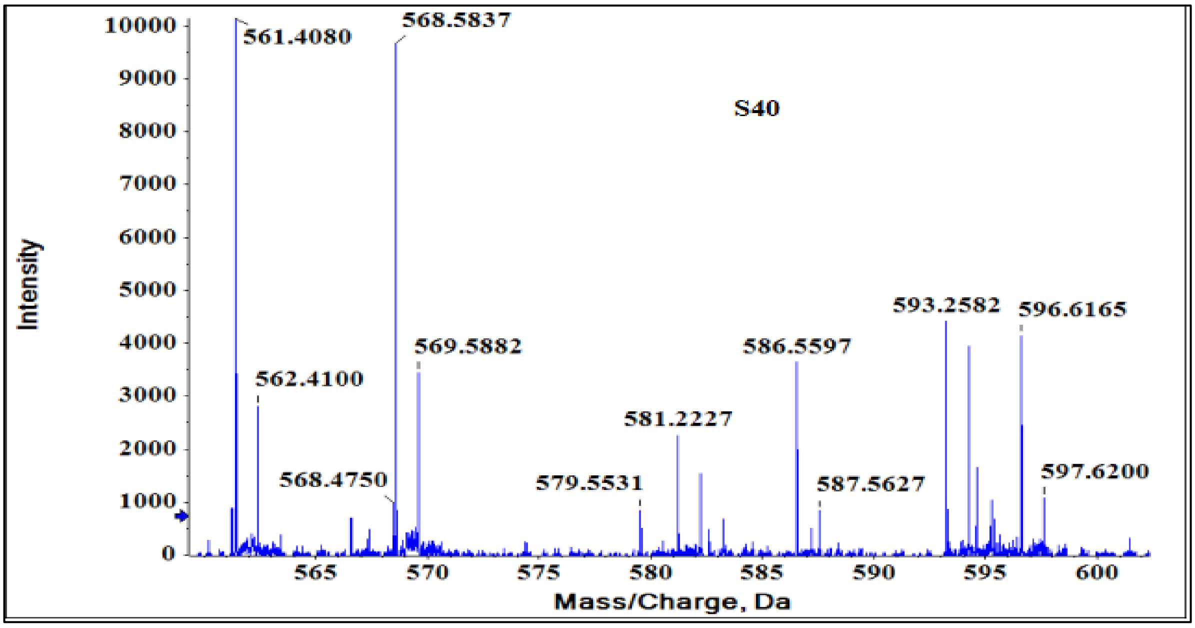

^{1}H NMR Spectra

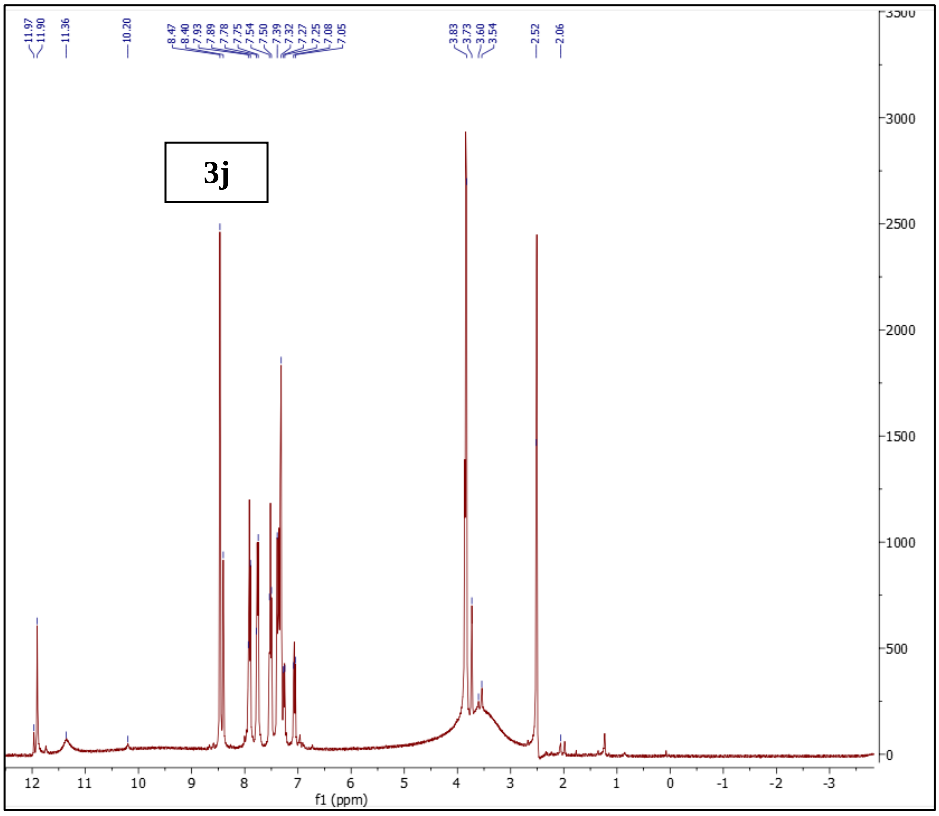

^{13}C NMR Spectra

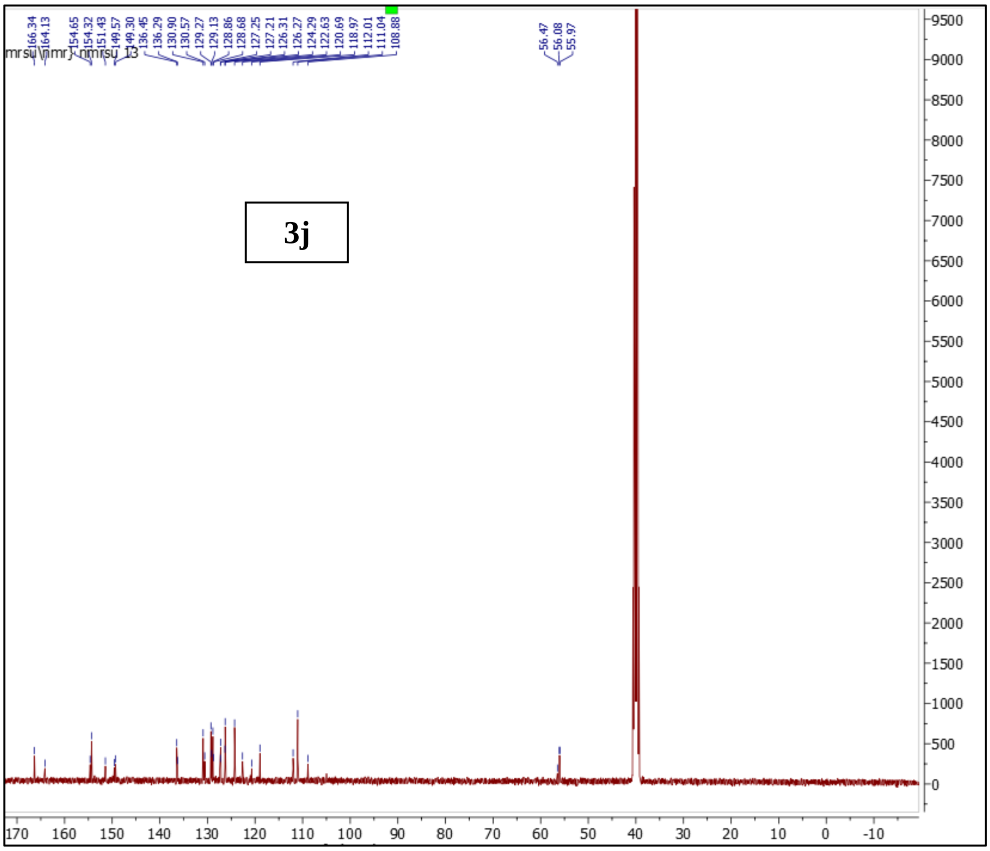

Supplement: Supplementary file 1 [file 49-6-683-supp.pdf]
